# Exploratory analysis of the 2-year changes in knee cartilage thickness and transverse relaxation time (T2) in ACL-injured versus healthy participants

**DOI:** 10.1016/j.ocarto.2026.100755

**Published:** 2026-02-05

**Authors:** Simon Herger, Wolfgang Wirth, Corina Nüesch, Oliver Bieri, Christian Egloff, Felix Eckstein, Annegret Mündermann

**Affiliations:** a Department of Orthopaedics and Traumatology, University Hospital Basel, Basel Switzerland; b Department of Biomedical Engineering, University of Basel, Allschwil, Switzerland; c Department of Clinical Research, University of Basel, Basel, Switzerland; d Department of Teaching, Research and Development, Schulthess Clinic, Zurich, Switzerland; e Research Program for Musculoskeletal Imaging, Center for Anatomy and Cell Biology & Ludwig Boltzmann Institute for Arthritis and Rehabilitation (LBIAR), Paracelsus Medical University, Salzburg, Austria; f Chondrometrics GmbH, Freilassing, Germany; g Department of Spine Surgery, University Hospital Basel, Basel, Switzerland; h Department of Radiology, University Hospital Basel, Basel, Switzerland; i Department of Hip and Knee Surgery, Schulthess Clinic, Zurich, Switzerland

**Keywords:** Posttraumatic osteoarthritis, Early osteoarthritis, Cartilage degeneration, Anterior cruciate ligament injury, qDESS, T2 mapping

## Abstract

**Objective:**

To investigate the 2-year changes in cartilage thickness and transverse relaxation time (T2) in magnetic resonance images (MRI) of ACL-injured (ACL_in) and uninjured contralateral knees (ACL_unin), compared to healthy controls (HEA).

**Method:**

Baseline and 2-year follow-up MRIs were acquired in both knees of 78 participants (ACL-injured 2–10 years prior, 20–30 years, n = 20; 40–60 years, n = 14; healthy, 20–30 years, n = 23; 40–60 years, n = 21). Weight bearing femorotibial cartilages were manually segmented to determine cartilage thickness and laminar (deep and superficial) T2. 2-year changes were compared between ACL_in, ACL_unin and HEA knees using estimated marginal means (EMM) and group differences in femorotibial compartments, (sub-)regions, and location-independent ordered values (OV).

**Results:**

From baseline to 2-year follow-up, cartilage thickness decreased in at least 50 % of regions/subregions whereas significant T2 changes ([95 % CI] ∉ 0) were rare. In ACL_in, cartilage EMM thinning (adjusted: MRI acquisition time, participant age) was strongest in the lateral tibia (LT) and its subregions (∼50 μm). In the interior LT subregion, thinning was 46 [70, 22], 46 [70, 22] and 13 [34, 8]μm in ACL_in, ACL_unin and HEA, respectively. In OV1 and OV2 of ACL_in, thinning was 178 [207, 150] and 130 [148, 111] μm, respectively and greater than in HEA. No EMM group differences were found for 2-year change in T2.

**Conclusions:**

ACL-injured knees displayed greater cartilage thinning, particularly using location-independent measures. Change in T2, in contrast, was limited and not specific to injury. Our findings underscore the need for relevant thresholds of cartilage thickness and T2 changes, to define early OA.

## 1 Introduction

Anterior cruciate ligament (ACL) tears are a frequent joint injury, with an annual incidence of 69 per 100′000 person-years and four fold higher rates in young persons between 14 and 25 years [1]. A recent meta-analysis reported an estimated prevalence of posttraumatic osteoarthritis (PTOA) of 36 % at 10 years after ACL surgery [2]. The development of PTOA after an ACL injury is thought to be driven by a combination of structural factors (high stress on articular cartilage and subchondral bone during trauma and joint instability), biological (disturbed cartilage metabolism), neuromuscular (reduced proprioception and muscle strength), and mechanical components (compromised knee stability and abnormal static and dynamic joint loading) [3]. The time point of ACL injury serves as a well-defined starting point, making it a useful model for studying early PTOA [3]. Given the high incidence of ACL injuries, particularly in young individuals, and the high prevalence of PTOA, many patients endure years of pain and reduced quality of life [4]. These factors underscore the need for early diagnosis (clinical practice) and classification (clinical research) of PTOA [5].

Although magnetic resonance imaging (MRI) is widely used to evaluate osteoarthritis (OA) [6], there is currently no established MRI-based definition for early OA [7,8], limiting its use in the detection of early PTOA. New MRI sequences such as the quantitative double echo steady state (qDESS) sequence enable simultaneous assessment of cartilage morphology (e.g., thickness, volume, surface areas) and composition (e.g., laminar (deep and superficial) transverse relaxation time (T2) mapping [5,9]). Compositional (quantitative) MRI has the potential to detect microstructural abnormalities and is therefore considered to be a valuable imaging biomarker for early OA [8]. T2 has been related to articular cartilage hydration, collagen content and orientation [10,11], and mechanical properties of the extracellular matrix [12]. Prolonged T2 has been reported in patients at risk for knee OA [13] and in fast progressors (Kellgren-Lawrence I to III within 5 years) before cartilage thinning became evident [14].

In a recent cross-sectional analysis, we showed with a manual [15] and an automated segmentation approach [16] that 2–10 years after ACL injury, cartilage T2 was prolonged in the injured knee compared to the contralateral uninjured and to healthy control knees primarily in the deep layer of the lateral femorotibial compartment, whereas no differences in cartilage thickness were observed. Longitudinal changes in femorotibial cartilage thickness have been reported in studies with follow-ups of 2 [17] or 5 [18,19] years after ACL injury. To date, only two studies investigated longitudinal change in both cartilage thickness and T2 both over a 2-year period after ACL reconstruction [20,21]. While both studies included healthy uninjured control knees at baseline, only Williams et al. [21] assessed longitudinal data also in controls. Neither study included the contralateral uninjured knee. Both studies reported longer T2 in the medial femur of ACL-injured knees compared to controls [20,21]. In addition, Su et al. [20] reported cartilage thickening in the medial femur and thinning in the lateral tibia, whereas Williams et al. [21] reported that early T2 changes (0–6 months) correlated with 2-year T2 changes, but not with cartilage thickness changes.

To date, longitudinal changes in cartilage thickness and T2 have rarely been examined simultaneously within the same study in ACL-injured knees, and have not been directly compared with corresponding changes in the contralateral or healthy knees. This study aimed to investigate longitudinal changes in cartilage thickness and laminar MRI T2 in the femorotibial joint after ACL injury over 2 years. We hypothesized that significant changes in thickness and T2 would occur in a greater number of regions and subregions and have a greater magnitude in ACL-injured knees than in the contralateral uninjured or than in healthy control knees.

## 2 Method

This specific analysis used data collected (January 2020 to August 2024) in an umbrella study [22] on the dose-response relationship between ambulatory load and load-induced cartilage biomarker kinetics. The umbrella study was approved by the regional ethics board (Ethics Committee Northwest and Central Switzerland EKNZ 2019-01315) and conducted in accordance with the Declaration of Helsinki. Participants provided written informed consent prior to participation.

### 2.1 Participants

Participants with a previous unilateral ACL injury 2–10 years prior to inclusion and participants without a knee injury were included in the umbrella study (n = 86). A complete list of inclusion and exclusion criteria for the umbrella study can be found in the published study protocol [22] and the recruitment strategy has been described previously [15]. A total of 85 participants had complete MRI data at baseline. Seven participants dropped out of the study (not reachable by phone or email, n = 4; personal reasons, n = 1; pregnancy, n = 1; elective leg lengthening surgery, n = 1). The remaining 78 participants in four groups (20–30 years ACL-injured, ACL_20–30_, n = 20; 20–30 years healthy, HEA_20–30_, n = 23; 40–60 years ACL-injured, ACL_40–60_, n = 14; 40–60 years healthy, HEA_40–60_, n = 21) completed 2-year follow-up data collection. Demographics and time between baseline and 2-year follow-up of the included participants are shown in Table 1.

**Table 1:** Participant characteristics (mean ± standard deviation).

Age group	20–30 years			40–60 years		
Injury group	ACL-injured N = 20	Healthy N = 23	SMD	ACL-injured N = 14	Healthy N = 21	SMD
Sex (female/male)	11/9	11/12		10/4	12/9	
Age (years)	28.2 ± 3.0	28.6 ± 3.1	−0.15	53.5 ± 5.5	51.3 ± 6.5	0.36
Body mass (kg)	72.0 ± 12.8	69.3 ± 10.8	0.22	68.2 ± 12.5	70.2 ± 11.4	−0.17
Height (cm)	172.8 ± 11.3	173.0 ± 8.7	−0.02	167.1 ± 10.6	172.6 ± 9.5	−0.56
BMI (kg/m^2^)	24.0 ± 2.6	23.1 ± 2.4	0.37	24.3 ± 2.6	23.7 ± 4.3	0.16
Time baseline to 2-year follow-up (months)	24.2 ± 1.0	24.9 ± 2.4	−0.36	23.9 ± 1.6	24.3 ± 0.7	−0.37
ACL injured knee (left/right)	10/10	*NA*		9/5	*NA*	
ACL injury treatment	Conservative: 2 surgical: 18 (ST: 16, PT: 2)	*NA*		Conservative: 3 surgical: 11 (ST: 10, PT: 1)	*NA*	
Time injury to baseline (months)	66.0 ± 34.4	*NA*		68.5 ± 33.3	*NA*	
Time injury to surgery (months)^a^	4.7 ± 7.0	*NA*		9.1 ± 17.1	*NA*	
Time surgery to baseline (months)^a^	61.9 ± 31.9	*NA*		62.7 ± 36.3	*NA*	

ACL—anterior cruciate ligament; SMD—standardized mean difference, BMI—body mass index; NA—not applicable; ST—Semitendinosus auto graft; PT—Patellar tendon auto graft;

a Variable computed for participants with surgical treatment only.

### 2.2 Procedures

These have been described previously [15]. In brief, after sitting still for 20–30 min, MRIs of both knees were acquired using a 3 T MR scanner (Prisma, Siemens Healthineers, Erlangen, Germany). A qDESS was acquired in a double oblique coronal plane (custom sequence, in-plane resolution 0.31 × 0.31 mm^2^, slice thickness 1.5 mm) [23]. The total acquisition time for both knees, including repositioning, was less than 60 min.

### 2.3 Image segmentation

MRI segmentation of ACL-injured (ACL_in) and uninjured (ACL_unin), left (HEA_l) and right (HEA_r) knees was performed manually by six experienced readers (>15 years) from Chondrometrics GmbH (Freilassing, Germany), with each subject's baseline and follow-up scans segmented by the same reader. Readers were blinded to ACL injury status and age, but not to scan order (baseline vs. follow-up). The femorotibial joint (FTJ) cartilage was segmented as described previously [24] using custom software [25] (accurate for thickness and T2 described in Refs. [26,27]). All segmentations were quality controlled by an expert reader, and corrections made by the original reader if needed.

**Fig. 1:**
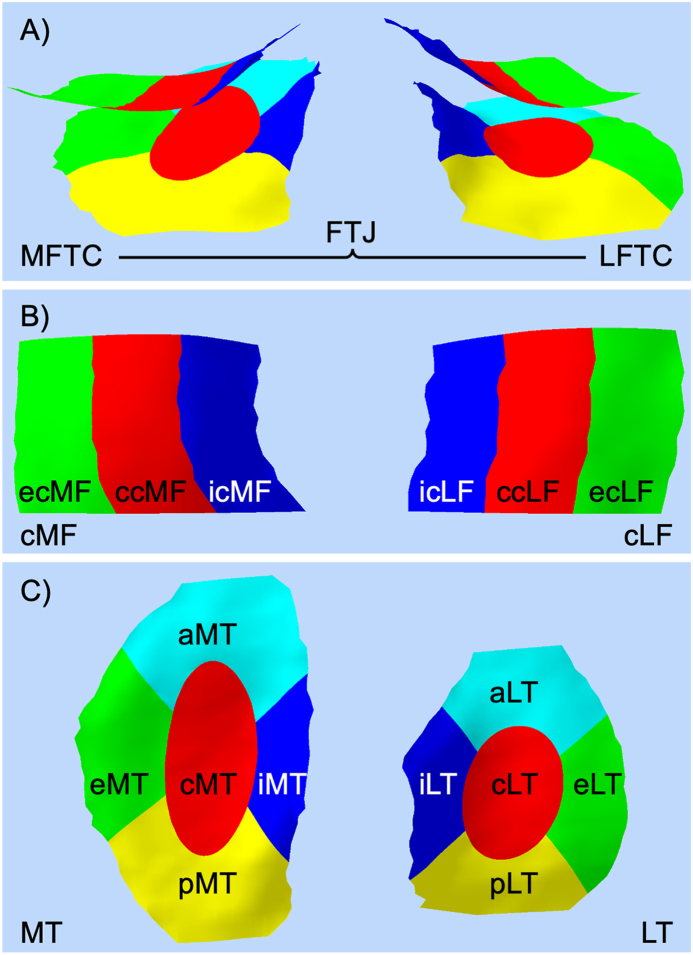
Illustration of the subregions in medial and lateral femorotibial compartment; MFTC—medial femorotibial compartment; LFTC—lateral femorotibial compartment; MT—medial tibia; cMF—central medial femur; LT—lateral tibia; cLF—central lateral femur; c—central; e—exterior; i—interior; a—anterior; p—posterior.

The mean cartilage thickness was first computed for the following regions: medial and lateral tibia (MT, LT) and weightbearing central medial and lateral femur (cMF, cLF). Next it was determined for exterior, central, and interior subregions (all four regions), and for anterior and posterior MT and LT subregions (described in Wirth et al. [25] and visualized in Fig. 1). Mean cartilage thickness was computed for the medial (MFTC, as MT + cMF) and for the lateral femorotibial compartment (LFTC, as LT + cLF), and for the FTJ ((MFTC + LFTC)/2).

For each voxel, T2 was estimated analytically from the two qDESS echoes according to the method developed by Heule et al. [23] and previously described in detail [15,16]. Local thickness was used to divide the cartilage into deep (lower 50 %) and superficial (upper 50 %) cartilage [25]. The mean T2 in the FTJ, the MFTC, and the LFTC was then calculated as the average of the respective regions (FTJ: MT, cMF, LT, and cLF; MFTC: MT and cMF, LFTC: LT and cLF).

### 2.4 Statistical analysis

All data were stored and managed using REDCap (Research Electronic Data Capture) [28,29] hosted at our orthopaedic clinic. Statistical analyses were performed with R (v4.3.1; R Core Team, Vienna, Austria) in RStudio (v2024.04.2; RStudio Team, Boston, MA, USA). The sample size calculation for the parent study was described in the study protocol [22]. Standardized mean differences (SMDs) were used to compare participant characteristics within the 20–30 and 40–60 year age groups (R package MBESS, 4.9.41).

The 2-year change in cartilage thickness and T2 for all compartments, regions, and subregions was computed as the mean at 2-year follow-up minus the mean at baseline. An ordered value (OV) approach was used [18,30] to assess location-independent subregional thickness changes across the FTJ. For each knee, the 2-year change in the 16 subregions was ranked based on the magnitude of change. OV1 and OV2 represent the subregions with the greatest and second greatest thinning, respectively, whereas OV16 and OV15 those with the greatest and second greatest thickening, respectively [18].

For all MRI parameters, the 2-year change did not differ between HEA_r and HEA_l, as assessed by paired t-tests after checking QQ plots for normality. Therefore, for subsequent analyses, we randomly selected either HEA_l or HEA_r, ensuring an equal number of left and right knees per age group and sex (hereafter referred to as HEA), to reduce the number of (parallel) statistical comparisons.

For each compartment, region and subregion, we computed a linear mixed effects model to estimate the mean 2-year change in cartilage thickness and T2 for ACL_in, ACL_unin, and HEA, allowing for a random intercept at the participant level (R package afex, 1.4-1). Estimated marginal means (EMMs) of the 2-year change and their 95 % confidence intervals (CI) were computed from these models (R package emmeans, 1.10.6.). Statistical significance for EMMs was determined based on the 95 % CI not including 0. Differences between ACL_in, ACL_unin, and HEA knees were assessed using pairwise comparisons of EMMs, Sidak-adjusted for multiple comparisons with significance set at 0.05. The influence of potential confounders was assessed prior to model building using a combination of scatter plots and univariate linear regression models (age, height, MRI acquisition time of day, body mass index (BMI) change, time from ACL injury to baseline MRI, corresponding baseline thickness or T2) (Figs. S1 and S2) and box plots (sex). Based on these assessments, cartilage thickness and T2 models were adjusted for participant age, differences in MRI acquisition time of day, and baseline thickness or T2 of corresponding compartment, region or subregion. Model assumptions (including normality of residuals and random effects, homogeneity of variance, and linearity) were confirmed visually (R package performance, v0.10.5).

## 3 Results

### 3.1 Participant characteristics

Descriptive statistics of participant characteristics are presented in Table 1.

### 3.2 2-Year change in knee cartilage thickness

The 2-year changes in knee cartilage thickness for all compartments, regions and subregions are presented as EMMs and their 95 % CI in Table 2. Of all four regions and 16 subregions, a statistically significant 2-year thinning of cartilage thickness was observed in 12 regions/subregions for ACL_in, 11 for ACL_unin, and 14 for HEA. In ACL_in, the strongest regional cartilage thinning (i.e., thickness decrease) occurred in the LT and its subregions eLT, iLT, and aLT, and, with a EMM thinning of 53 μm, 47 μm, 46 μm, and 56 μm, respectively (relative to baseline 2.4 %, 2.9 %, 2.1 % and 3.3 %, respectively). In ACL_unin, the strongest cartilage thinning occurred in the icLF, iLT, and cMT (49 μm, 46 μm, and 43 μm, respectively; relative to baseline 3.0 %, 2.1 %, 1.8 %, respectively). In HEA, cartilage thinning was most pronounced in the cLF and its subregions ccLF and ecLF (43 μm, 45 μm, and 49 μm, respectively; relative to baseline 2.6 %, 2.3 %, 3.4 %, respectively). No statistically significant cartilage thickening was observed in any of the analyzed compartments, regions, or subregions in ACL_in, ACL_unin or HEA (Table 2).

**Table 2:** Estimated marginal means [95 % CI] of 2-year changes in knee cartilage thickness and estimated marginal mean group differences (μm).

	ACL_in	ACL_unin	HEA	ACL_in - ACL_unin	ACL_in - HEA	ACL_unin - HEA
**Compartments**						
FTJ	**−58 [-86, -29]**	**−59 [-87, -31]**	**−62 [-86, -37]**	1 [-29, 32]	4 [-33, 41]	3 [-34, 39]
MFTC	−31 [-63, 2]	**−55 [-88, -23]**	**−50 [-79, -22]**	25 [-17, 66]	20 [-23, 62]	−5 [-47, 37]
LFTC	**−84 [-117, -51]**	**−63 [-96, -30]**	**−73 [-102, -44]**	−21 [-60, 18]	−11 [-55, 33]	10 [-34, 53]
**Regions**						
MT	**−24 [-41, -6]**	**−35 [-52, -18]**	**−22 [-37, -7]**	11 [-6, 29]	−2 [-25, 21]	−13 [-35, 10]
cMF	−6 [-30, 17]	−21 [-44, 3]	**−29 [-49, -8]**	15 [-18, 47]	23 [-8, 53]	8 [-22, 39]
LT	**−53 [-74, -32]**	**−31 [-52, -10]**	**−31 [-49, -12]**	−22 [-52, 8]	−22 [-50, 5]	0 [-28, 27]
cLF	**−30 [-52, -8]**	**−32 [-54, -11]**	**−43 [-62, -24]**	2 [-27, 30]	13 [-16, 42]	11 [-17, 39]
**Subregions**						
cMT	−12 [-42, 19]	**−43 [-73, -12]**	**−29 [-56, -2]**	31 [-1, 63]'	17 [-23, 58]	−14 [-53, 26]
eMT	−13 [-38, 12]	**−28 [-54, -3]**	−20 [-42, 2]	15 [-13, 44]	7 [-26, 39]	−9 [-41, 24]
iMT	**−39 [-59, -18]**	**−37 [-57, -16]**	**−24 [-42, -6]**	−2 [-30, 27]	−15 [-41, 12]	−13 [-40, 14]
aMT	**−34 [-61, -6]**	**−32 [-59, -4]**	**−25 [-49, -1]**	−2 [-38, 34]	−9 [-45, 28]	−7 [-42, 29]
pMT	−19 [-41, 2]	**−38 [-59, -16]**	−14 [-33, 5]	18 [-9, 46]	−6 [-34, 22]	−24 [-52, 4]
ccMF	−2 [-38, 34]	−15 [-51, 21]	**−36 [-68, -4]**	13 [-36, 62]	34 [-14, 81]	21 [-26, 68]
ecMF	21 [-4, 47]	−6 [-31, 19]	**−23 [-45, -1]**	27 [-5, 59]	**44 [11, 78]∗∗**	17 [-16, 50]
icMF	**−32 [-59, -6]**	**−39 [-65, -12]**	**−28 [-51, -5]**	7 [-31, 44]	−4 [-39, 31]	−11 [-45, 24]
cLT	**−75 [-116, -34]**	−35 [-76, 6]	−33 [-69, 3]	−40 [-98, 18]	−42 [-95, 12]	−1 [-55, 52]
eLT	**−47 [-71, -23]**	−18 [-42, 6]	**−39 [-60, -18]**	−29 [-60, 2]'	−8 [-40, 24]	21 [-11, 52]
iLT	**−46 [-70, -22]**	**−46 [-70, -22]**	−13 [-34, 8]	1 [-33, 34]	**−33 [-64, -1]∗**	**−33 [-65, -2]∗**
aLT	**−56 [-89, -22]**	−21 [-55, 12]	−27 [-57, 2]	−34 [-77, 8]	−29 [-72, 15]	6 [-38, 49]
pLT	−40 [-85, 6]	−35 [-81, 11]	−38 [-78, 2]	−4 [-69, 60]	−1 [-61, 58]	3 [-56, 63]
ccLF	**−33 [-63, -4]**	−29 [-57, 0]	**−45 [-71, -19]**	−5 [-42, 33]	12 [-28, 51]	17 [-22, 55]
ecLF	−19 [-44, 6]	−17 [-42, 7]	**−49 [-71, -27]**	−1 [-33, 30]	30 [-3, 63]'	31 [-1, 64]'
icLF	**−37 [-64, -9]**	**−49 [-76, -22]**	**−38 [-62, -14]**	12 [-26, 51]	2 [-34, 38]	−11 [-46, 25]
**Location-independent subregional changes**						
OV1	**−179 [-207, -150]**	**−149 [-177, -121]**	**−136 [-160, -111]**	−30 [-70, 10]	**−43 [-80, -6]∗**	−13 [-50, 24]
OV2	**−130 [-148, -111]**	**−112 [-131, -94]**	**−98 [-114, -82]**	−18 [-40, 5]	**−32 [-56, -7]∗∗**	−14 [-38, 10]
OV15	**52 [32, 72]**	**54 [34, 74]**	**40 [22, 57]**	−2 [-26, 23]	13 [-14, 39]	14 [-12, 40]
OV16	**87 [59, 114]**	**87 [60, 115]**	**76 [51, 100]**	−1 [-32, 31]	11 [-25, 47]	12 [-24, 48]

ACL—anterior cruciate ligament; ACL_in—ACL-injured; ACL_unin—ACL-uninjured; HEA—healthy; FTJ—femorotibial joint; MFTC—medial femorotibial compartment; LFTC—lateral femorotibial compartment; MT—medial tibia; cMF—central medial femur; LT—lateral tibia; cLF—central lateral femur; c—central; e—exterior; i—interior; a—anterior; p—posterior; OV1 and OV2—greatest and second greatest location-independent subregional decrease in thickness; OV16 and OV15—greatest and second greatest location-independent subregional increase in thickness; all models used to compute EMMs adjusted for participant age, differences in MRI acquisition daytime, and corresponding compartmental, regional or subregional baseline thickness. Significant 2-year changes and group differences in bold, Sidak-adjusted group differences: ∗p < 0.05, ∗∗p < 0.01.

Among the subregions with significant cartilage thinning, the EMM change in iLT was significantly more pronounced in ACL_in and ACL_unin than in HEA, with a stronger thinning of 33 μm (effect size: 0.58, 95 % CI [0.10, 1.05]) and 33 μm (effect size: 0.59, 95 % CI [0.11, 1.06]), respectively (Table 2). In ecMF, the 21 μm cartilage thickness increase observed in ACL_in (not statistically different from 0) was 44 μm greater (effect size: 0.85, 95 % CI [0.31, 1.39]) than the significant thinning of 23 μm observed in HEA. No other relevant group differences in 2-year cartilage thickness change between ACL_in, ACL_unin, and HEA were observed in other compartments, regions, or subregions.

The strongest location-independent cartilage thinning was observed in ACL_in (179 μm, 130 μm), followed by ACL_unin (149 μm, 112 μm) and HEA (136 μm, 98 μm), in OV1 and OV2, respectively (Table 2). Cartilage thinning was stronger in ACL_in compared with HEA in OV1 (EMM difference, 43 μm; effect size: 0.65, 95 % CI [1.12, 0.18]) and OV2 (32 μm; effect size: 0.85, 95 % CI [1.40, 0.30]) (Table 2).

Location-independent cartilage thickening was similar in ACL_in (EMM, 87 μm, 52 μm), in ACL_unin (87 μm, 54 μm) and in HEA (76 μm, 40 μm) for OV16 and OV15, respectively, and did not statistically differ between ACL_in, ACL_unin, and HEA (Table 2). An overview of subregions included in the OVs are presented in Table 3.

**Table 3:** Number of occurrences of each subregion to corresponding ordered value (OV) as a count and relative to number of subjects per group (%).

	OV16			OV15			OV2			OV1		
	ACL_in	ACL_unin	HEA	ACL_in	ACL_unin	HEA	ACL_in	ACL_unin	HEA	ACL_in	ACL_unin	HEA
cMT	3 (8.8)	0 (0)	**5 (11.4)**	3 (8.8)	3 (8.8)	**4 (9.1)**	1 (2.9)	**2 (5.9)**	3 (6.8)	1 (2.9)	2 (5.9)	4 (9.1)
eMT	**4 (11.8)**	1 (2.9)	1 (2.3)	1 (2.9)	**7 (20.6)**	2 (4.5)	1 (2.9)	**2 (5.9)**	1 (2.3)	2 (5.9)	2 (5.9)	0 (0)
iMT	1 (2.9)	2 (5.9)	1 (2.3)	1 (2.9)	1 (2.9)	3 (6.8)	1 (2.9)	**2 (5.9)**	2 (4.5)	0 (0)	1 (2.9)	0 (0)
aMT	2 (5.9)	4 (11.8)	4 (9.1)	2 (5.9)	0 (0)	2 (4.5)	**5 (14.7)**	**3 (8.8)**	1 (2.3)	1 (2.9)	0 (0)	4 (9.1)
pMT	0 (0)	2 (5.9)	2 (4.5)	2 (5.9)	1 (2.9)	3 (6.8)	0 (0)	1 (2.9)	0 (0)	1 (2.9)	1 (2.9)	0 (0)
ccMF	**4 (11.8)**	**6 (17.6)**	2 (4.5)	3 (8.8)	**4 (11.8)**	3 (6.8)	**4 (11.8)**	1 (2.9)	4 (9.1)	2 (5.9)	**4 (11.8)**	4 (9.1)
ecMF	**4 (11.8)**	4 (11.8)	4 (9.1)	**8 (23.5)**	2 (5.9)	1 (2.3)	0 (0)	**3 (8.8)**	2 (4.5)	0 (0)	0 (0)	1 (2.3)
icMF	1 (2.9)	0 (0)	1 (2.3)	1 (2.9)	0 (0)	1 (2.3)	**4 (11.8)**	1 (2.9**)**	1 (2.3)	2 (5.9)	**4 (11.8)**	2 (4.5)
cLT	1 (2.9)	4 (11.8)	3 (6.8)	1 (2.9)	3 (8.8)	**6 (13.6)**	2 (5.9)	**2 (5.9)**	1 (2.3)	**8 (23.5)**	**6 (17.6)**	**5 (11.4)**
eLT	1 (2.9)	1 (2.9)	2 (4.5)	1 (2.9)	3 (8.8)	2 (4.5)	1 (2.9)	**3 (8.8)**	**5 (11.4)**	2 (5.9)	0 (0)	4 (9.1)
iLT	2 (5.9)	0 (0)	4 (9.1)	0 (0)	1 (2.9)	**6 (13.6)**	3 (8.8)	**3 (8.8)**	4 (9.1)	1 (2.9)	2 (5.9)	0 (0)
aLT	3 (8.8)	3 (8.8)	**5 (11.4)**	1 (2.9)	2 (5.9)	1 (2.3)	2 (5.9)	1 (2.9)	2 (4.5)	**5 (14.7)**	1 (2.9)	2 (4.5)
pLT	**5 (14.7)**	**5 (14.7)**	**6 (13.6)**	4 (11.8)	2 (5.9)	3 (6.8)	**5 (14.7)**	**3 (8.8)**	**8 (18.2)**	**5 (14.7)**	**6 (17.6)**	**7 (15.9)**
ccLF	1 (2.9)	1 (2.9)	0 (0)	0 (0)	3 (8.8)	**4 (9.1)**	1 (2.9)	**3 (8.8)**	3 (6.8)	1 (2.9)	2 (5.9)	4 (9.1)
ecLF	1 (2.9)	1 (2.9)	3 (6.8)	**5 (14.7)**	1 (2.9)	2 (4.5)	2 (5.9)	**2 (5.9)**	4 (9.1)	2 (5.9)	0 (0)	**5 (11.4)**
icLF	1 (2.9)	0 (0)	1 (2.3)	1 (2.9)	1 (2.9)	1 (2.3)	2 (5.9)	**2 (5.9)**	3 (6.8)	1 (2.9)	3 (8.8)	2 (4.5)

ACL—anterior cruciate ligament; ACL_in—ACL-injured; ACL_unin—ACL-uninjured; HEA—healthy; MT—medial tibia; cMF—central medial femur; LT—lateral tibia; cLF—central lateral femur; c—central; e—exterior; i—interior; a—anterior; p—posterior; OV1 and OV2—greatest and second greatest location-independent subregional decrease in thickness; OV16 and OV15—greatest and second greatest location-independent subregional increase in thickness; bold underlined—subregion(s) with the highest number of occurrences in corresponding OV; bold— subregion(s) with the 2nd highest number of occurrences in corresponding OV.

### 3.3 Knee cartilage T2

The 2-year changes in T2 for all compartments and regions are presented as EMMs and their 95 % CI in Table 4. Among the four regions with three layers (total, deep, and superficial), a significant 2-year increase in T2 was observed in two layer-region combinations for ACL_in, two for ACL_unin, and two for HEA (Table 4). In ACL_in, an increase in EMM T2 was observed in the MT and LT deep zone (0.7 ms, and 0.8 ms, respectively; relative to baseline 3.5 %, and 3.7 %, respectively). In ACL_unin, the strongest T2 increases were found in the total and superficial LT total (1.1 ms, and 1.5 ms, respectively; relative to baseline 3.7 %, and 3.9 %). In HEA, the strongest T2 increases were found in total and superficial cMF (1.0 ms, and 1.6 ms, respectively; relative to baseline 2.6 %, and 3.2 %, respectively). No statistically significant 2-year EMM decrease in T2 was observed in any of the analyzed compartments and regions. Further, no significant differences in the EMM of the 2-year change in T2 were found between ACL_in, ACL_unin, and HEA (Table 4).

**Table 4:** Estimated marginal means [95 % CI] of 2-year changes in knee cartilage T2 and estimated marginal mean group differences (ms).

	ACL_in	ACL_unin	HEA	ACL_in - ACL_unin	ACL_in - HEA	ACL_unin - HEA
**Compartments**						
FTJ.T	0.4 [-0.3, 1.1]	0.6 [-0.1, 1.4]	**0.6 [0.0, 1.3]**	−0.2 [-1.0, 0.5]	−0.2 [-1.2, 0.7]	0.0 [-0.9, 0.9]
FTJ.D	**0.7 [0.2, 1.2]**	0.4 [-0.1, 0.9]	0.1 [-0.3, 0.6]	0.3 [-0.4, 1.1]	0.6 [-0.1, 1.3]	0.2 [-0.4, 0.9]
FTJ.S	0.2 [-0.8, 1.3]	0.8 [-0.2, 1.8]	**1.0 [0.1, 1.9]**	−0.6 [-1.6, 0.5]	−0.7 [-2.1, 0.6]	−0.2 [-1.5, 1.2]
MFTC.T	0.4 [-0.5, 1.2]	0.4 [-0.5, 1.2]	0.6 [-0.2, 1.3]	0.0 [-0.9, 0.9]	−0.2 [-1.3, 0.9]	−0.2 [-1.3, 0.9]
MFTC.D	**0.7 [0.1, 1.2]**	0.4 [-0.1, 0.9]	0.2 [-0.3, 0.6]	0.3 [-0.4, 1.0]	0.5 [-0.2, 1.2]	0.2 [-0.5, 0.9]
MFTC.S	0.2 [-1.1, 1.5]	0.3 [-1.0, 1.5]	0.8 [-0.3, 1.9]	−0.1 [-1.4, 1.3]	−0.6 [-2.2, 1.1]	−0.5 [-2.2, 1.1]
LFTC.T	0.4 [-0.4, 1.2]	**0.9 [0.1, 1.7]**	**0.7 [0.0, 1.4]**	−0.5 [-1.4, 0.5]	−0.3 [-1.3, 0.8]	0.2 [-0.8, 1.2]
LFTC.D	**0.7 [0.0, 1.3]**	0.4 [-0.2, 0.9]	0.1 [-0.4, 0.6]	0.3 [-0.5, 1.2]	0.6 [-0.3, 1.5]	0.3 [-0.5, 1.0]
LFTC.S	0.3 [-0.9, 1.4]	**1.3 [0.1, 2.5]**	**1.1 [0.1, 2.2]**	−1.0 [-2.3, 0.2]	−0.8 [-2.4, 0.7]	0.2 [-1.3, 1.7]
**Regions**						
MT.T	0.5 [-0.3, 1.4]	0.4 [-0.4, 1.3]	0.1 [-0.6, 0.9]	0.1 [-0.9, 1.1]	0.4 [-0.7, 1.5]	0.3 [-0.8, 1.4]
MT.D	**0.7 [0.2, 1.2]**	**0.5 [0.1, 1.0]**	0.2 [-0.2, 0.6]	0.2 [-0.4, 0.7]	0.5 [-0.1, 1.1]	0.3 [-0.3, 0.9]
MT.S	0.4 [-1.0, 1.7]	0.3 [-1.0, 1.6]	0.0 [-1.2, 1.2]	0.1 [-1.5, 1.7]	0.4 [-1.4, 2.1]	0.3 [-1.4, 2.1]
cMF.T	0.2 [-0.9, 1.3]	0.3 [-0.8, 1.4]	**1.0 [0.0, 1.9]**	−0.2 [-1.3, 1.0]	−0.8 [-2.3, 0.6]	−0.7 [-2.1, 0.8]
cMF.D	0.6 [-0.2, 1.5]	0.3 [-0.6, 1.1]	0.1 [-0.6, 0.8]	0.4 [-0.8, 1.5]	0.5 [-0.6, 1.6]	0.2 [-0.9, 1.3]
cMF.S	0.0 [-1.6, 1.6]	0.3 [-1.4, 1.9]	**1.6 [0.2, 3.0]**	−0.3 [-2.0, 1.5]	−1.6 [-3.7, 0.5]	−1.3 [-3.5, 0.8]
LT.T	0.5 [-0.3, 1.3]	**1.1 [0.3, 1.8]**	0.5 [-0.1, 1.2]	−0.5 [-1.6, 0.5]	0.0 [-1.1, 1.0]	0.5 [-0.5, 1.5]
LT.D	**0.8 [0.1, 1.5]**	0.6 [-0.1, 1.2]	0.3 [-0.3, 0.9]	0.2 [-0.7, 1.1]	0.5 [-0.4, 1.4]	0.3 [-0.6, 1.2]
LT.S	0.3 [-0.9, 1.4]	**1.5 [0.3, 2.6]**	0.8 [-0.2, 1.8]	−1.2 [-2.7, 0.3]	−0.5 [-2.0, 0.9]	0.7 [-0.8, 2.1]
cLF.T	0.3 [-0.8, 1.5]	0.7 [-0.4, 1.9]	0.9 [-0.1, 1.8]	−0.4 [-1.9, 1.1]	−0.5 [-2.0, 1.0]	−0.1 [-1.6, 1.4]
cLF.D	0.7 [-0.2, 1.5]	0.1 [-0.7, 0.9]	−0.1 [-0.8, 0.6]	0.6 [-0.6, 1.7]	0.8 [-0.3, 1.9]	0.2 [-0.8, 1.2]
cLF.S	0.3 [-1.5, 2.0]	1.1 [-0.6, 2.9]	1.5 [0.0, 3.0]	−0.9 [-3.0, 1.2]	−1.2 [-3.5, 1.0]	−0.4 [-2.6, 1.9]

ACL—anterior cruciate ligament; ACL_in—ACL-injured; ACL_unin—ACL-uninjured; HEA—healthy; FTJ—femorotibial joint; T—total T2; D—deep zone T2; S—superficial zone T2; MFTC—medial femorotibial compartment; LFTC—lateral femorotibial compartment; MT—medial tibia; cMF—central medial femur; LT—lateral tibia; cLF—central lateral femur; all models used to compute EMMs adjusted for participant age, differences in MRI acquisition daytime, and corresponding compartmental or regional baseline T2, Significant 2-year changes and group differences in bold, Sidak-adjusted group differences: ∗p < 0.05, ∗∗p < 0.01.

Absolute baseline, 2-year follow-up values and 2-year changes for cartilage thickness and T2 are reported in Supplementary Tables S1 and S2, respectively.

## 4 Discussion

Our first hypothesis, that 2-year changes in cartilage thickness and T2 occur in a greater number of regions, and subregions in ACL-injured knees compared to the contralateral uninjured knee and healthy controls, had to be refuted. While the number of regions and subregions with significant 2-year cartilage thickness thinning was similar and around 50 % among ACL-injured, contralateral uninjured, and healthy control knees, a significant 2-year increase in T2 were observed in fewer region-layer combinations in patients 2–10 years post-ACL injury compared to contralateral uninjured and healthy control knees. Our second hypothesis that the 2-year changes would have a greater magnitude after ACL injury was confirmed for location-independent cartilage thinning (OV1 and OV2) and location-specific thinning in iLT, but not for other location-specific changes in thickness or T2.

Over the 2 years, we observed a decrease in FTJ cartilage thickness of ∼60 μm in the ACL-injured, uninjured knee and healthy control knees. This contrasts with Eckstein et al. [18], who reported an annual increase in FTJ thickness of 31 μm over 5 years [18], and Su et al. [20], who found that cartilage thickness in the lateral tibia was generally lower in ACL-injured participants than in healthy controls and showed an increase in thickness from baseline to 2 years after ACL injury. In both studies, baseline MRI was acquired within a maximum of 28 [18] or 46 days (before ACL reconstruction) [20] of ACL rupture, whereas our baseline MRI were acquired after a mean of more than 5 years after ACL injury. Our results suggest a cartilage thinning in the LFTC over the 2-year period in individuals after ACL injury. This thinning was most pronounced in the LT region, especially in the iLT subregion, and occurred in both the ACL-injured and contralateral uninjured knee where thinning was significantly greater than in control knees.

Although cartilage thinning after ACL injury was most pronounced in subregions of the lateral tibia, only the iLT subregion showed low enough within-group variability in both the ACL-injured and contralateral knees to detect a 2-year change significantly different from the change observed in healthy controls. The variability observed in subregional cartilage thinning may reflect the interindividual differences in the location of the most pronounced cartilage deterioration. The ordered value approach offers the advantage of neglecting heterogeneity in the patient specific location of the greatest thinning or thickening by summarizing the greatest subregional 2-year changes in single variables. In OV1, 2-year cartilage thickness decreased by 178.7 μm, 148.8 μm and 135.5 μm in the ACL-injured, uninjured knee and healthy control knees, respectively. It is not surprising that the magnitude of location-independent changes observed exceed the previously described subregional location-specific changes. The observed cartilage thinning in OV1 and OV2 was significantly greater in the ACL-injured knees than the change observed in healthy knees, with an even greater effect size in OV2. This implies that ACL-injured knees experienced more substantial cartilage loss than healthy knees not only in the region with the greatest thinning (OV1) but also in the region with the second greatest thinning (OV2). Our observed 2-year changes in OV1 and OV2 where greater than the annual thinning of 48 μm in OV1 reported by Eckstein et al. [18] over the first 5 years after ACL injury. The greater 2-year changes in our participants more than 5 years after ACL-injury contrast with their findings, which reported higher OV1 thinning of 115 μm in the early follow-up period (baseline to year 2), compared to the thinning of 54 μm in later follow-up period (year 2–5) [18]. To better understand the initial increase in cartilages thickness (swelling) and subsequent thinning (degradation of cartilage) following an ACL injury, longitudinal studies starting immediately after the trauma and including follow-ups longer than 5 years and a wider range of age groups are necessary. The observed longitudinal thickness changes in the ACL-injured, but also in the contralateral ACL-uninjured knee further supports the concept that the contralateral ACL-uninjured knee does not represent a completely “healthy control” knee [31]. Therefore, we encourage the inclusion of healthy controls without history of knee injuries in future longitudinal studies.

In line with these findings, we observed no meaningful association between 2-year changes in cartilage thickness and corresponding changes in T2, and this lack of association was consistent across ACL-injured, contralateral uninjured, and healthy control knees (Fig. S4). T2 relaxation time changed over 2 years only in a few region-layer combinations. T2 is a quantitative measure related to cartilage collagen content and orientation [10,11]. Further factors influencing T2 are water content [32], cartilage loading [33], cartilage orientation relative to the magnetic field due to the magic angle effect [34]. Since all these factors can influence T2, longitudinal changes in T2 relaxation time may be challenging to interpret [6].

Nonetheless, it is notable that the prolonged deep zone T2 time in ACL-injured knees compared to contralateral uninjured knees and healthy controls observed in our baseline data [15,16] persisted at 2-year follow-up (Table S2). A recent study by Toguchi et al. [35], reported prolonged T2 values in the medial and lateral tibia as early as 3 months and 1 year after acute ACL injury in comparison to a control group. Similarly, other studies in individuals after ACL injury reported absolute T2 at multiple timepoints over 9 months [35] or 2 years [20], but did not compute longitudinal T2 changes and compared them to T2 changes observed in control knees.

This is one of the first studies reporting both longitudinal cartilage thickness and T2 changes for ACL-injured and additionally the contralateral uninjured and healthy control knees. T2 increased in the total and superficial zone of the lateral tibia in contralateral knees and in the superficial central lateral and medial femur of healthy control knees over 2 years. Similar to our study, Pedoia et al. [36] assessed longitudinal 6-month and 1-year changes after ACL-reconstruction and the contralateral ACL-uninjured knees (n = 40) and 1-year changes in healthy control knees (n = 15). They reported an increase in T2 at 6 months after ACL reconstruction in ACL-injured but also in the contralateral uninjured knee. Further, they reported a T2 increase over one year in the medial and lateral tibia of healthy controls [36]. In our study, we also observed a longitudinal change in medial and lateral femoral superficial T2 of healthy control knees. However, the relevance of these longitudinal T2 changes in early OA diagnosis and classification are poorly understood. Rather than relying on statistical significance, it would be necessary to define thresholds (e.g., % of baseline or absolute values) for clinically meaningful longitudinal changes in T2. This would simplify and strengthen the use of longitudinal T2 changes in the early diagnosis and classification of OA.

The main strengths of our study include the highly standardized MRI acquisition protocol, with baseline and 2-year follow-up images obtained by the same two technicians. In addition, participants were restricted to walk for 30 min prior to MRI, high spatial resolution qDESS was used, bilateral assessments were performed, and a large number of healthy controls that were included. A limitation of this study is that no participants between 30 and 40 years of age were included, due to the grouped study design of the umbrella study [22]. Further, reported cartilage changes over a 2-year follow-up are relatively small (<4 %), which approaches the limits of measurement precision, reported as root-mean-square coefficient of variation ∼1–3% for cartilage thickness [37,38] and root-mean-square-average coefficient of variation <5 % for T2 [39]. Although readers were unblinded to scan order, a recent study in OA patients showed a high correlation between blinded and unblinded readings, but also that scan order unblinded analysis can introduce systematic bias [40]. Since the same procedure was applied for ACL injured and healthy participants, we expect minimal effects on group comparisons.

To counteract potential age effects, statistical models examining 2-year changes in thickness and T2 were adjusted for participant age. In addition, it was not possible for baseline and 2-year follow-up MRI to be performed at the same time of the day in one participant. To account for possible time of day effects [41], the models for change in thickness and T2 were adjusted for within-participant differences in MRI acquisition time of day between baseline and 2-year follow-up but not for potential within-participant variability in the 20–30 min pre-MRI sitting time. Although, this study included subjects 2–10 years after ACL injury, the models were not adjusted for the varying time after ACL injury because neither visual inspection of scatterplots nor univariate linear regression analyses (e.g., slopes not significant different from zero, Figs. S1 and S2) did indicate any systematic effect of the time after ACL injury on change in thickness or T2. However, time after ACL injury, especially for cartilage thickness (Fig. S3), is reflected in the adjustment for baseline values. Although small changes in cartilage thickness can slightly alter the probability of voxel assignment between superficial and deep layers, the large number of voxel stacks (>9000) makes such effects unlikely to substantially affect mean laminar T2 values. Total cartilage T2 values (unaffected by laminar classification) are presented in Table 4 and S2. It should further be noted that although MRI readers were formally blinded to ACL injury status, they could potentially infer injury status based on imaging characteristics such as surgical tunnels or metallic artifact from fixation devices. For T2, OV analysis was not feasible because subregional mean values from coronal MRI were not available at the time of segmentation.

Because there is no clear consensus on the risk of PTOA following surgical or conservative treatment [2,42,43], ACL-injured participants were included regardless of the treatment type. Although most participants underwent ACL reconstruction with a semitendinosus autograft, we did not include this as covariate due to the small number of cases for each treatment. Our participants were on average more than 5 years after ACL injury at baseline and were followed for a period of 2 years, and our analysis focused on the comparison between ACL-injured, ACL-uninjured and healthy control knees. We did not consider intraindividual differences in the stage or rate of cartilage degeneration, the localization of degeneration (related to different injury mechanisms or altered biomechanics in the years after trauma) nor associations to bone marrow lesions. Therefore, our data do not allow to elucidate longitudinal thickness and T2 changes over a period longer than 2 years and/or on an individual level, which may be important to gain valuable insights into the pathophysiology of PTOA.

## 5 Conclusion

Our hypothesis that ACL-injured knees would show greater cartilage thinning and T2 lengthening over 2 years was only partially supported. While location-independent thickness measures (OV1, OV2) indicated more pronounced cartilage thinning in ACL-injured knees, the number of regions and subregions with significant thickness changes was similar in ACL-injured, contralateral ACL-uninjured, and healthy control knees. Surprisingly, the number of regions and layers with significant 2-year changes in T2 was even smaller in ACL-injured knees compared to controls. However, the elevated deep zone T2 values observed at baseline in ACL-injured knees persisted from baseline to 2-year follow-up, suggesting pre-existing cartilage composition changes in cartilage compositions. The presence of T2 changes in both contralateral and healthy control knees highlight the need for clinically meaningful thresholds to better interpret longitudinal T2 changes and their relevance in early detection and classification of OA.

## Author contributions

SH, AM, and CE designed the study; AM and CE obtained funding for the study; SH recruited the participants, collected and processed the data, and conducted the statistical analysis; SH, WW, FE, CN, and AM designed the statistical analysis; SH drafted the manuscript and visualized the data; all authors interpreted the data, critically reviewed the manuscript, contributed to the revision of the manuscript, and agreed on the final draft.

## Conflict of interest

The authors declare no conflict of interest.
