# Supplementary material for: Exploratory analysis of the 2-year changes in knee cartilage thickness and transverse relaxation time (T2) in ACL-injured versus healthy participants
Source: Osteoarthr Cartil Open. 2026 Feb 5;8(1):100755. doi: 10.1016/j.ocarto.2026.100755 (PMC12936675; doi:10.1016/j.ocarto.2026.100755)
Supplement: Multimedia component 1 [file mmc1.docx]

Figure S1:


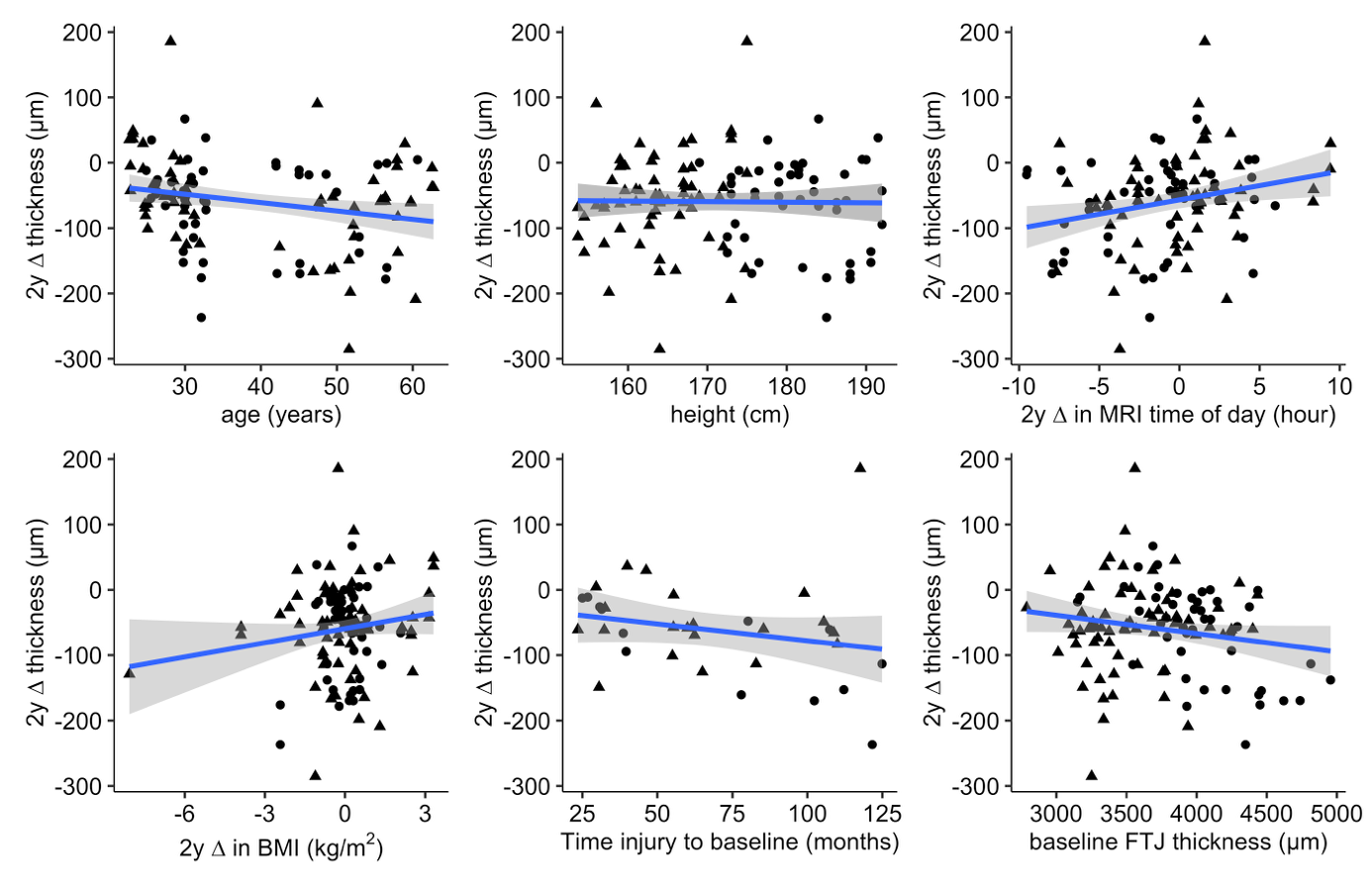


Figure 1: Scatterplots showing the associations between participant characteristics and 2-year change (2y ∆) in femorotibial joint (FTJ) cartilage thickness. Each point represents an individual knee; circles indicate male participants and triangles indicate female participants. Solid blue lines represent linear regression fits with 95% confidence intervals (grey shading).

Figure S2:


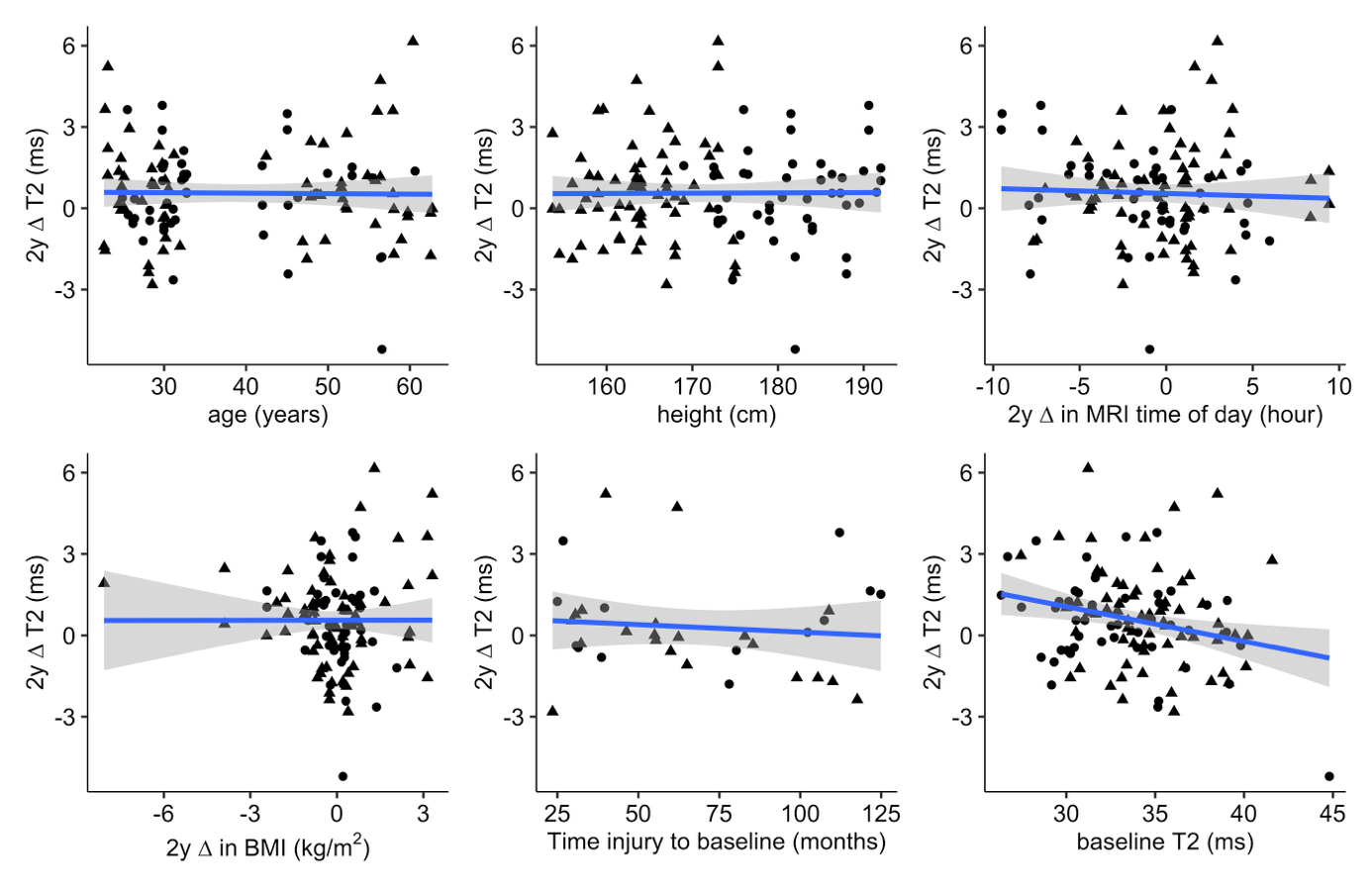


Figure 2: Scatterplots showing the associations between participant characteristics and 2-year change (2y ∆) in femorotibial joint (FTJ) total T2. Each point represents an individual knee; circles indicate male participants and triangles indicate female participants. Solid blue lines represent linear regression fits with 95% confidence intervals (grey shading).

Figure S3:


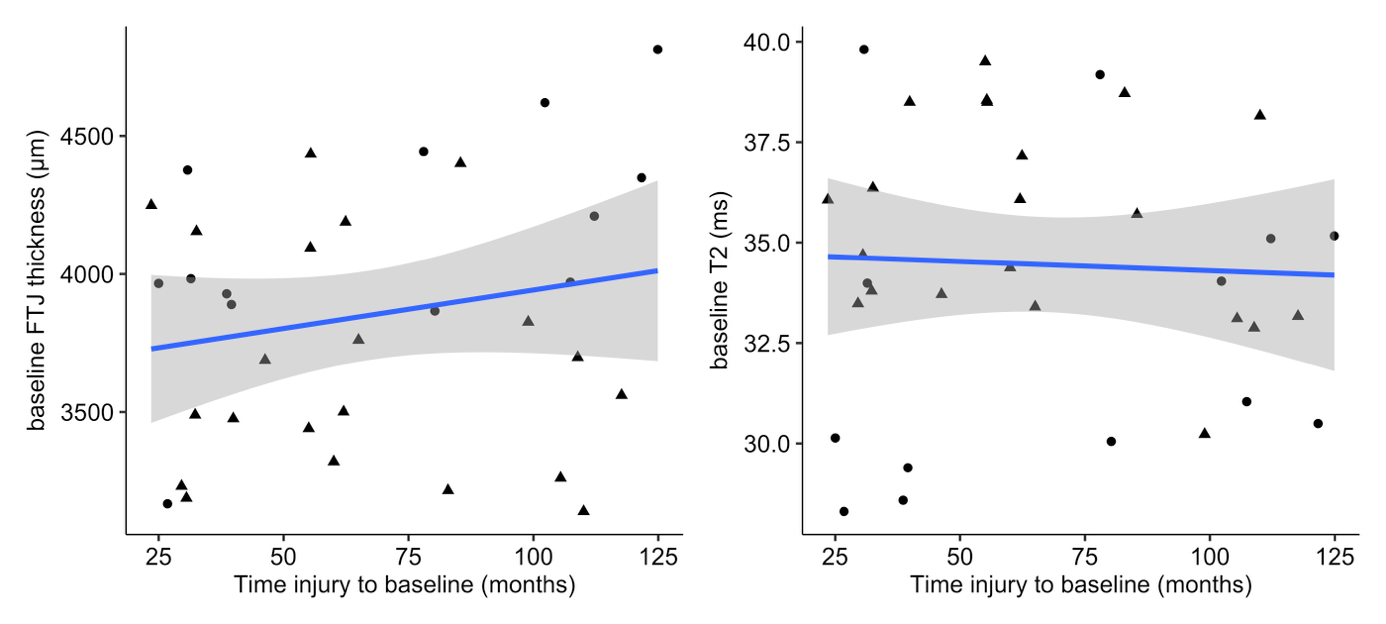


Figure 3: Associations between time from ACL injury to baseline MRI and baseline FTJ cartilage thickness (left) and total T2 values (right). Circles indicate males; triangles indicate females. Blue lines show linear fits with 95% confidence intervals.

Figure S4:


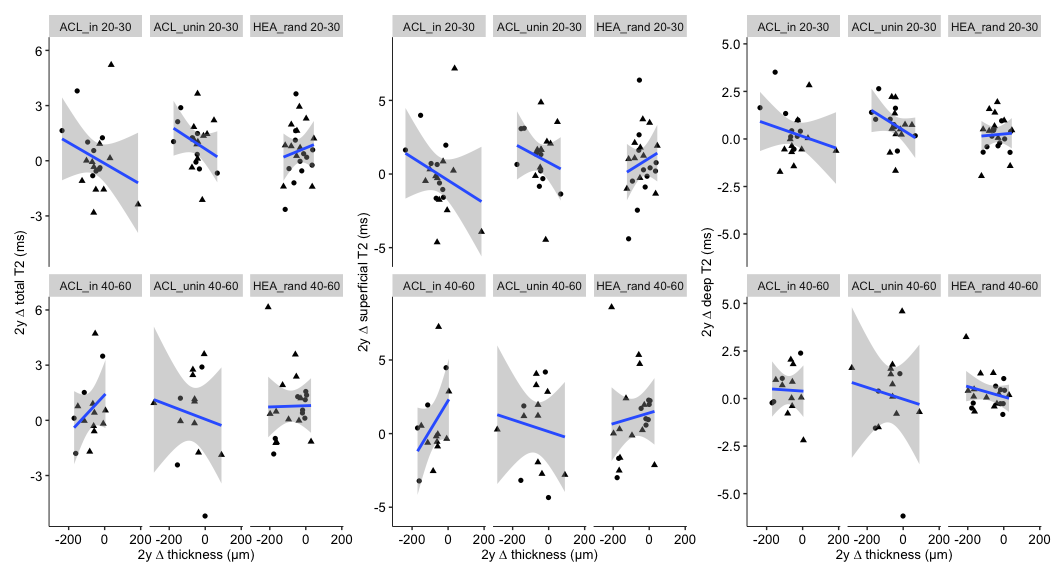


Figure 4: Scatterplots showing associations between 2-year changes (2y ∆) in cartilage thickness and T2 relaxation times (total, superficial, deep). Panels display ACL-injured (ACL_in), contralateral uninjured (ACL_unin), and healthy control (HEA_rand) groups, stratified by age (20–30 and 40–60 years). Circles indicate males; triangles indicate females. Blue lines indicate linear regression with 95% confidence intervals in grey.
