# Supplementary material for: Exploratory analysis of the 2-year changes in knee cartilage thickness and transverse relaxation time (T2) in ACL-injured versus healthy participants
Source: Osteoarthr Cartil Open. 2026 Feb 5;8(1):100755. doi: 10.1016/j.ocarto.2026.100755 (PMC12936675; doi:10.1016/j.ocarto.2026.100755)
Supplement: Multimedia component 2 [file mmc2.docx]

Table S1: Absolute values of cartilage thickness at baseline, 2-year follow-up and thickness changes (µm) for subgroups of the study

|  | **20–30 years** | | | | | | | | |  | **40–60 years** | | | | | | | | |
| --- | --- | --- | --- | --- | --- | --- | --- | --- | --- | --- | --- | --- | --- | --- | --- | --- | --- | --- | --- |
|  | **ACL-injured** | | | | |  | **Healthy** | | |  | **ACL-injured** | | | | |  | **Healthy** | | |
|  | **female** | |  | **male** | |  | **female** |  | **male** |  | **female** | |  | **male** | |  | **female** |  | **male** |
|  | **ACL_in** | **ACL_unin** |  | **ACL_in** | **ACL_unin** |  | **HEA** |  | **HEA** |  | **ACL_in** | **ACL_unin** |  | **ACL_in** | **ACL_unin** |  | **HEA** |  | **HEA** |
| **n** | 11 | 11 |  | 9 | 9 |  | 11 |  | 12 |  | 10 | 10 |  | 4 | 4 |  | 12 |  | 9 |
| **Compartments** | | | | | | | | | | | | | | | | | | | |
| Baseline FTJ | 3818 ± 378 | 3653 ± 332 |  | 4060 ± 198 | 3898 ± 260 |  | 3546 ± 234 |  | 3822 ± 332 |  | 3531 ± 431 | 3457 ± 252 |  | 4261 ± 745 | 4251 ± 771 |  | 3302 ± 326 |  | 4071 ± 282 |
| 2-year FTJ | 3796 ± 365 | 3622 ± 345 |  | 3979 ± 174 | 3827 ± 211 |  | 3502 ± 242 |  | 3786 ± 319 |  | 3467 ± 454 | 3388 ± 295 |  | 4148 ± 683 | 4173 ± 727 |  | 3196 ± 272 |  | 4023 ± 250 |
| Change FTJ | -22 ± 85 | -31 ± 36 |  | -81 ± 72 | -71 ± 74 |  | -44 ± 55 |  | -36 ± 47 |  | -64 ± 45 | -69 ± 96 |  | -114 ± 73 | -78 ± 79 |  | -106 ± 76 |  | -48 ± 73 |
| Baseline MFTC | 3546 ± 433 | 3431 ± 344 |  | 3942 ± 381 | 3701 ± 348 |  | 3337 ± 259 |  | 3630 ± 369 |  | 3372 ± 460 | 3262 ± 322 |  | 4195 ± 798 | 4052 ± 925 |  | 3180 ± 308 |  | 3915 ± 410 |
| 2-year MFTC | 3552 ± 422 | 3409 ± 355 |  | 3876 ± 334 | 3642 ± 309 |  | 3310 ± 263 |  | 3599 ± 351 |  | 3346 ± 482 | 3180 ± 355 |  | 4088 ± 690 | 3997 ± 886 |  | 3094 ± 269 |  | 3871 ± 364 |
| Change MFTC | 6 ± 78 | -22 ± 44 |  | -66 ± 67 | -59 ± 72 |  | -27 ± 84 |  | -31 ± 71 |  | -26 ± 73 | -82 ± 85 |  | -108 ± 140 | -55 ± 107 |  | -86 ± 86 |  | -44 ± 93 |
| Baseline LFTC | 4091 ± 378 | 3875 ± 349 |  | 4177 ± 337 | 4095 ± 328 |  | 3755 ± 266 |  | 4014 ± 351 |  | 3690 ± 421 | 3653 ± 246 |  | 4327 ± 716 | 4450 ± 643 |  | 3424 ± 383 |  | 4228 ± 291 |
| 2-year LFTC | 4040 ± 366 | 3835 ± 371 |  | 4082 ± 312 | 4011 ± 304 |  | 3694 ± 264 |  | 3973 ± 348 |  | 3587 ± 450 | 3597 ± 279 |  | 4207 ± 697 | 4350 ± 602 |  | 3298 ± 332 |  | 4176 ± 280 |
| Change LFTC | -51 ± 106 | -40 ± 56 |  | -95 ± 103 | -83 ± 107 |  | -61 ± 50 |  | -41 ± 41 |  | -103 ± 62 | -56 ± 123 |  | -120 ± 53 | -101 ± 56 |  | -126 ± 75 |  | -52 ± 70 |
| **Regions** | | | | | | | | | | | | | | | | | | | |
| Baseline MT | 1693 ± 188 | 1631 ± 159 |  | 1862 ± 153 | 1736 ± 132 |  | 1606 ± 134 |  | 1703 ± 193 |  | 1667 ± 188 | 1613 ± 160 |  | 2017 ± 348 | 2027 ± 425 |  | 1568 ± 151 |  | 1953 ± 191 |
| 2-year MT | 1689 ± 174 | 1609 ± 160 |  | 1822 ± 132 | 1711 ± 134 |  | 1597 ± 135 |  | 1701 ± 186 |  | 1634 ± 198 | 1566 ± 176 |  | 1979 ± 285 | 1973 ± 424 |  | 1517 ± 129 |  | 1933 ± 169 |
| Change MT | -4 ± 40 | -22 ± 24 |  | -39 ± 38 | -24 ± 56 |  | -10 ± 31 |  | -2 ± 33 |  | -33 ± 36 | -47 ± 39 |  | -38 ± 69 | -53 ± 30 |  | -51 ± 42 |  | -20 ± 62 |
| Baseline cMF | 1853 ± 268 | 1800 ± 222 |  | 2080 ± 281 | 1966 ± 268 |  | 1731 ± 177 |  | 1927 ± 205 |  | 1705 ± 285 | 1649 ± 187 |  | 2179 ± 454 | 2025 ± 505 |  | 1612 ± 199 |  | 1961 ± 269 |
| 2-year cMF | 1863 ± 279 | 1801 ± 219 |  | 2053 ± 248 | 1931 ± 252 |  | 1714 ± 164 |  | 1898 ± 200 |  | 1713 ± 299 | 1613 ± 206 |  | 2109 ± 407 | 2023 ± 472 |  | 1577 ± 174 |  | 1938 ± 251 |
| Change cMF | 10 ± 61 | 0 ± 59 |  | -27 ± 57 | -35 ± 44 |  | -17 ± 65 |  | -29 ± 47 |  | 8 ± 50 | -35 ± 60 |  | -70 ± 79 | -2 ± 113 |  | -35 ± 53 |  | -24 ± 37 |
| Baseline LT | 2277 ± 227 | 2177 ± 235 |  | 2275 ± 171 | 2250 ± 207 |  | 2163 ± 197 |  | 2240 ± 202 |  | 2042 ± 155 | 2067 ± 148 |  | 2336 ± 312 | 2485 ± 345 |  | 1927 ± 244 |  | 2384 ± 202 |
| 2-year LT | 2249 ± 206 | 2166 ± 254 |  | 2207 ± 159 | 2208 ± 193 |  | 2133 ± 174 |  | 2239 ± 198 |  | 1972 ± 169 | 2046 ± 192 |  | 2284 ± 299 | 2400 ± 322 |  | 1869 ± 199 |  | 2355 ± 214 |
| Change LT | -27 ± 49 | -11 ± 38 |  | -68 ± 58 | -43 ± 56 |  | -30 ± 39 |  | -1 ± 25 |  | -70 ± 43 | -22 ± 87 |  | -53 ± 44 | -85 ± 47 |  | -58 ± 55 |  | -29 ± 39 |
| Baseline cLF | 1814 ± 220 | 1698 ± 177 |  | 1902 ± 230 | 1844 ± 172 |  | 1592 ± 194 |  | 1774 ± 222 |  | 1649 ± 307 | 1585 ± 144 |  | 1991 ± 415 | 1965 ± 304 |  | 1497 ± 186 |  | 1844 ± 159 |
| 2-year cLF | 1790 ± 228 | 1669 ± 172 |  | 1875 ± 228 | 1803 ± 160 |  | 1561 ± 184 |  | 1734 ± 219 |  | 1616 ± 311 | 1551 ± 153 |  | 1924 ± 416 | 1950 ± 284 |  | 1430 ± 176 |  | 1821 ± 130 |
| Change cLF | -24 ± 80 | -29 ± 66 |  | -27 ± 59 | -41 ± 54 |  | -31 ± 49 |  | -39 ± 21 |  | -33 ± 41 | -34 ± 53 |  | -67 ± 12 | -15 ± 78 |  | -67 ± 39 |  | -23 ± 45 |
| **Subregions** | | | | | | | | | | | | | | | | | | | |
| Baseline cMT | 2443 ± 303 | 2250 ± 244 |  | 2737 ± 230 | 2483 ± 234 |  | 2168 ± 224 |  | 2363 ± 370 |  | 2283 ± 283 | 2220 ± 184 |  | 2965 ± 717 | 3000 ± 774 |  | 2162 ± 306 |  | 2828 ± 364 |
| 2-year cMT | 2460 ± 322 | 2237 ± 253 |  | 2689 ± 236 | 2447 ± 266 |  | 2157 ± 227 |  | 2376 ± 347 |  | 2269 ± 292 | 2157 ± 219 |  | 2922 ± 625 | 2927 ± 781 |  | 2100 ± 267 |  | 2777 ± 322 |
| Change cMT | 17 ± 70 | -12 ± 28 |  | -48 ± 61 | -37 ± 108 |  | -11 ± 68 |  | 13 ± 75 |  | -14 ± 60 | -63 ± 64 |  | -43 ± 115 | -74 ± 41 |  | -62 ± 86 |  | -51 ± 83 |
| Baseline eMT | 1411 ± 182 | 1373 ± 146 |  | 1588 ± 174 | 1467 ± 99 |  | 1368 ± 137 |  | 1403 ± 182 |  | 1347 ± 154 | 1322 ± 230 |  | 1668 ± 255 | 1703 ± 318 |  | 1325 ± 160 |  | 1639 ± 257 |
| 2-year eMT | 1416 ± 155 | 1347 ± 130 |  | 1546 ± 159 | 1457 ± 106 |  | 1351 ± 128 |  | 1392 ± 169 |  | 1329 ± 176 | 1286 ± 221 |  | 1659 ± 196 | 1655 ± 318 |  | 1285 ± 133 |  | 1636 ± 245 |
| Change eMT | 5 ± 88 | -25 ± 59 |  | -42 ± 51 | -10 ± 84 |  | -17 ± 54 |  | -11 ± 45 |  | -18 ± 62 | -36 ± 78 |  | -9 ± 83 | -48 ± 27 |  | -39 ± 47 |  | -4 ± 57 |
| Baseline iMT | 1743 ± 200 | 1771 ± 253 |  | 2077 ± 248 | 1929 ± 248 |  | 1713 ± 186 |  | 1860 ± 226 |  | 1868 ± 240 | 1779 ± 213 |  | 2095 ± 300 | 2169 ± 410 |  | 1734 ± 252 |  | 2150 ± 212 |
| 2-year iMT | 1727 ± 190 | 1726 ± 253 |  | 2024 ± 239 | 1913 ± 239 |  | 1707 ± 184 |  | 1845 ± 215 |  | 1818 ± 232 | 1742 ± 195 |  | 2042 ± 291 | 2109 ± 376 |  | 1681 ± 250 |  | 2132 ± 215 |
| Change iMT | -16 ± 55 | -45 ± 42 |  | -53 ± 68 | -16 ± 68 |  | -5 ± 46 |  | -14 ± 37 |  | -50 ± 35 | -36 ± 40 |  | -53 ± 12 | -61 ± 56 |  | -53 ± 43 |  | -18 ± 71 |
| Baseline aMT | 1472 ± 185 | 1362 ± 128 |  | 1760 ± 133 | 1571 ± 134 |  | 1379 ± 135 |  | 1523 ± 220 |  | 1536 ± 220 | 1453 ± 153 |  | 1761 ± 367 | 1814 ± 355 |  | 1365 ± 206 |  | 1721 ± 282 |
| 2-year aMT | 1444 ± 177 | 1350 ± 136 |  | 1735 ± 103 | 1553 ± 132 |  | 1357 ± 128 |  | 1526 ± 228 |  | 1463 ± 202 | 1404 ± 200 |  | 1760 ± 259 | 1752 ± 335 |  | 1304 ± 192 |  | 1712 ± 243 |
| Change aMT | -28 ± 78 | -12 ± 69 |  | -25 ± 75 | -18 ± 58 |  | -22 ± 26 |  | 4 ± 56 |  | -73 ± 55 | -48 ± 56 |  | -1 ± 114 | -62 ± 56 |  | -61 ± 55 |  | -9 ± 95 |
| Baseline pMT | 1468 ± 193 | 1484 ± 131 |  | 1299 ± 218 | 1343 ± 223 |  | 1473 ± 188 |  | 1452 ± 187 |  | 1394 ± 192 | 1371 ± 218 |  | 1697 ± 191 | 1620 ± 388 |  | 1341 ± 160 |  | 1567 ± 131 |
| 2-year pMT | 1474 ± 198 | 1459 ± 128 |  | 1266 ± 223 | 1309 ± 202 |  | 1475 ± 200 |  | 1448 ± 184 |  | 1385 ± 211 | 1324 ± 204 |  | 1617 ± 188 | 1567 ± 373 |  | 1306 ± 130 |  | 1546 ± 121 |
| Change pMT | 6 ± 47 | -25 ± 27 |  | -33 ± 29 | -33 ± 111 |  | 2 ± 52 |  | -4 ± 42 |  | -9 ± 48 | -48 ± 50 |  | -80 ± 47 | -54 ± 16 |  | -35 ± 47 |  | -21 ± 47 |
| Baseline ccMF | 2302 ± 358 | 2235 ± 287 |  | 2690 ± 607 | 2512 ± 512 |  | 2065 ± 245 |  | 2374 ± 272 |  | 2032 ± 359 | 2003 ± 266 |  | 2718 ± 542 | 2531 ± 654 |  | 1909 ± 248 |  | 2436 ± 449 |
| 2-year ccMF | 2300 ± 406 | 2249 ± 296 |  | 2680 ± 564 | 2491 ± 496 |  | 2045 ± 216 |  | 2334 ± 289 |  | 2055 ± 378 | 1963 ± 282 |  | 2626 ± 486 | 2512 ± 585 |  | 1872 ± 225 |  | 2406 ± 425 |
| Change ccMF | -2 ± 112 | 14 ± 100 |  | -10 ± 83 | -21 ± 61 |  | -20 ± 98 |  | -41 ± 81 |  | 24 ± 68 | -41 ± 97 |  | -92 ± 79 | -19 ± 153 |  | -37 ± 78 |  | -30 ± 55 |
| Baseline ecMF | 1397 ± 250 | 1348 ± 176 |  | 1488 ± 165 | 1361 ± 105 |  | 1265 ± 141 |  | 1368 ± 196 |  | 1268 ± 361 | 1222 ± 163 |  | 1641 ± 286 | 1433 ± 263 |  | 1220 ± 161 |  | 1372 ± 244 |
| 2-year ecMF | 1440 ± 242 | 1345 ± 206 |  | 1475 ± 143 | 1350 ± 85 |  | 1252 ± 111 |  | 1340 ± 195 |  | 1303 ± 349 | 1211 ± 165 |  | 1591 ± 264 | 1458 ± 235 |  | 1183 ± 137 |  | 1376 ± 225 |
| Change ecMF | 43 ± 59 | -3 ± 73 |  | -13 ± 59 | -11 ± 45 |  | -13 ± 65 |  | -27 ± 53 |  | 35 ± 52 | -11 ± 55 |  | -49 ± 45 | 25 ± 119 |  | -36 ± 46 |  | 5 ± 78 |
| Baseline icMF | 1880 ± 245 | 1833 ± 258 |  | 2086 ± 216 | 2036 ± 240 |  | 1857 ± 261 |  | 2043 ± 221 |  | 1819 ± 255 | 1727 ± 222 |  | 2197 ± 535 | 2112 ± 607 |  | 1708 ± 230 |  | 2079 ± 166 |
| 2-year icMF | 1872 ± 253 | 1825 ± 251 |  | 2031 ± 228 | 1967 ± 231 |  | 1838 ± 257 |  | 2023 ± 211 |  | 1790 ± 277 | 1674 ± 243 |  | 2131 ± 475 | 2102 ± 596 |  | 1674 ± 206 |  | 2037 ± 162 |
| Change icMF | -7 ± 70 | -8 ± 66 |  | -56 ± 67 | -69 ± 68 |  | -20 ± 62 |  | -20 ± 54 |  | -30 ± 61 | -52 ± 55 |  | -67 ± 116 | -10 ± 80 |  | -34 ± 68 |  | -42 ± 45 |
| Baseline cLT | 3471 ± 389 | 3271 ± 442 |  | 3618 ± 397 | 3452 ± 396 |  | 3375 ± 427 |  | 3480 ± 443 |  | 3264 ± 245 | 3254 ± 286 |  | 3845 ± 658 | 4091 ± 659 |  | 3002 ± 610 |  | 3752 ± 398 |
| 2-year cLT | 3421 ± 384 | 3277 ± 489 |  | 3565 ± 397 | 3404 ± 382 |  | 3323 ± 401 |  | 3475 ± 452 |  | 3125 ± 219 | 3235 ± 369 |  | 3794 ± 580 | 3940 ± 681 |  | 2942 ± 523 |  | 3746 ± 428 |
| Change cLT | -51 ± 90 | 6 ± 89 |  | -53 ± 72 | -48 ± 101 |  | -52 ± 95 |  | -5 ± 38 |  | -138 ± 97 | -19 ± 163 |  | -50 ± 117 | -151 ± 51 |  | -61 ± 111 |  | -6 ± 84 |
| Baseline eLT | 1738 ± 206 | 1698 ± 159 |  | 1707 ± 220 | 1666 ± 177 |  | 1604 ± 220 |  | 1695 ± 228 |  | 1464 ± 206 | 1450 ± 188 |  | 1725 ± 299 | 1932 ± 254 |  | 1428 ± 190 |  | 1825 ± 157 |
| 2-year eLT | 1703 ± 188 | 1687 ± 189 |  | 1666 ± 194 | 1664 ± 175 |  | 1567 ± 186 |  | 1682 ± 222 |  | 1412 ± 225 | 1438 ± 189 |  | 1640 ± 309 | 1839 ± 253 |  | 1372 ± 184 |  | 1773 ± 140 |
| Change eLT | -36 ± 48 | -11 ± 51 |  | -40 ± 60 | -2 ± 62 |  | -37 ± 70 |  | -13 ± 47 |  | -52 ± 63 | -12 ± 105 |  | -86 ± 45 | -93 ± 13 |  | -56 ± 34 |  | -52 ± 58 |
| Baseline iLT | 2174 ± 281 | 2147 ± 277 |  | 2340 ± 229 | 2305 ± 197 |  | 2121 ± 246 |  | 2251 ± 258 |  | 1955 ± 221 | 2006 ± 194 |  | 2293 ± 308 | 2258 ± 327 |  | 1850 ± 282 |  | 2211 ± 275 |
| 2-year iLT | 2155 ± 284 | 2108 ± 299 |  | 2270 ± 222 | 2262 ± 217 |  | 2117 ± 224 |  | 2260 ± 263 |  | 1903 ± 189 | 1962 ± 211 |  | 2238 ± 290 | 2177 ± 320 |  | 1803 ± 232 |  | 2206 ± 304 |
| Change iLT | -19 ± 30 | -39 ± 67 |  | -70 ± 52 | -43 ± 40 |  | -4 ± 59 |  | 9 ± 60 |  | -53 ± 72 | -45 ± 78 |  | -55 ± 37 | -81 ± 70 |  | -47 ± 70 |  | -5 ± 49 |
| Baseline aLT | 1675 ± 251 | 1606 ± 147 |  | 1749 ± 230 | 1707 ± 303 |  | 1581 ± 144 |  | 1702 ± 189 |  | 1611 ± 230 | 1639 ± 188 |  | 1793 ± 282 | 1872 ± 308 |  | 1529 ± 176 |  | 1902 ± 227 |
| 2-year aLT | 1619 ± 198 | 1603 ± 184 |  | 1689 ± 203 | 1676 ± 296 |  | 1548 ± 163 |  | 1702 ± 181 |  | 1546 ± 240 | 1632 ± 224 |  | 1761 ± 197 | 1790 ± 270 |  | 1478 ± 146 |  | 1881 ± 236 |
| Change aLT | -56 ± 141 | -3 ± 71 |  | -60 ± 119 | -32 ± 95 |  | -33 ± 47 |  | 0 ± 69 |  | -64 ± 65 | -7 ± 65 |  | -32 ± 98 | -82 ± 39 |  | -51 ± 53 |  | -21 ± 77 |
| Baseline pLT | 2412 ± 226 | 2247 ± 349 |  | 2050 ± 264 | 2194 ± 209 |  | 2201 ± 206 |  | 2150 ± 238 |  | 1967 ± 243 | 2042 ± 286 |  | 2115 ± 239 | 2377 ± 415 |  | 1869 ± 210 |  | 2293 ± 286 |
| 2-year pLT | 2439 ± 264 | 2240 ± 344 |  | 1942 ± 310 | 2112 ± 134 |  | 2182 ± 190 |  | 2153 ± 210 |  | 1920 ± 279 | 2014 ± 287 |  | 2065 ± 332 | 2342 ± 373 |  | 1788 ± 190 |  | 2236 ± 293 |
| Change pLT | 27 ± 139 | -7 ± 58 |  | -108 ± 82 | -82 ± 95 |  | -18 ± 99 |  | 3 ± 76 |  | -47 ± 180 | -27 ± 101 |  | -50 ± 164 | -35 ± 113 |  | -81 ± 89 |  | -57 ± 63 |
| Baseline ccLF | 2280 ± 289 | 2060 ± 249 |  | 2352 ± 381 | 2232 ± 220 |  | 1855 ± 272 |  | 2097 ± 303 |  | 2002 ± 393 | 1893 ± 175 |  | 2430 ± 502 | 2423 ± 345 |  | 1777 ± 274 |  | 2304 ± 204 |
| 2-year ccLF | 2252 ± 302 | 2049 ± 223 |  | 2311 ± 393 | 2184 ± 197 |  | 1839 ± 253 |  | 2061 ± 295 |  | 1963 ± 403 | 1857 ± 189 |  | 2374 ± 463 | 2411 ± 313 |  | 1702 ± 251 |  | 2270 ± 157 |
| Change ccLF | -28 ± 91 | -11 ± 97 |  | -41 ± 79 | -49 ± 68 |  | -15 ± 67 |  | -36 ± 41 |  | -40 ± 51 | -36 ± 79 |  | -56 ± 42 | -12 ± 100 |  | -75 ± 67 |  | -35 ± 65 |
| Baseline ecLF | 1631 ± 221 | 1485 ± 193 |  | 1615 ± 344 | 1620 ± 229 |  | 1436 ± 159 |  | 1601 ± 232 |  | 1426 ± 384 | 1399 ± 169 |  | 1639 ± 480 | 1682 ± 203 |  | 1274 ± 155 |  | 1510 ± 163 |
| 2-year ecLF | 1599 ± 226 | 1473 ± 226 |  | 1608 ± 364 | 1594 ± 236 |  | 1386 ± 165 |  | 1536 ± 235 |  | 1411 ± 371 | 1379 ± 170 |  | 1609 ± 466 | 1672 ± 207 |  | 1228 ± 147 |  | 1488 ± 165 |
| Change ecLF | -32 ± 90 | -12 ± 61 |  | -7 ± 62 | -26 ± 66 |  | -50 ± 69 |  | -66 ± 25 |  | -15 ± 55 | -20 ± 61 |  | -30 ± 25 | -10 ± 91 |  | -46 ± 37 |  | -23 ± 56 |
| Baseline icLF | 1566 ± 233 | 1574 ± 181 |  | 1761 ± 200 | 1702 ± 184 |  | 1502 ± 192 |  | 1643 ± 190 |  | 1541 ± 208 | 1485 ± 181 |  | 1931 ± 367 | 1820 ± 372 |  | 1455 ± 176 |  | 1744 ± 160 |
| 2-year icLF | 1555 ± 237 | 1512 ± 159 |  | 1728 ± 157 | 1655 ± 184 |  | 1474 ± 179 |  | 1626 ± 180 |  | 1498 ± 221 | 1439 ± 179 |  | 1818 ± 433 | 1798 ± 351 |  | 1373 ± 157 |  | 1729 ± 123 |
| Change icLF | -12 ± 90 | -62 ± 97 |  | -33 ± 67 | -47 ± 50 |  | -28 ± 61 |  | -17 ± 33 |  | -43 ± 55 | -46 ± 59 |  | -113 ± 68 | -22 ± 66 |  | -82 ± 50 |  | -14 ± 51 |

ACL—anterior cruciate ligament; ACL_in—ACL-injured; ACL_unin—ACL-uninjured; HEA—healthy; FTJ—femorotibial joint; MFTC—medial femorotibial compartment; LFTC—lateral femorotibial compartment; MT—medial tibia; cMF—central medial femur; LT—lateral tibia; cLF—central lateral femur; c—central; e—exterior; i—interior; a—anterior; p—posterior;
