# Supplementary material for: Exploratory analysis of the 2-year changes in knee cartilage thickness and transverse relaxation time (T2) in ACL-injured versus healthy participants
Source: Osteoarthr Cartil Open. 2026 Feb 5;8(1):100755. doi: 10.1016/j.ocarto.2026.100755 (PMC12936675; doi:10.1016/j.ocarto.2026.100755)
Supplement: Multimedia component 3 [file mmc3.docx]

Table S2: Absolute values of cartilage transverse relaxation time (T2) at baseline, 2-year follow-up and T2 changes for subgroups of the study (ms)

|  | **20–30 years** | | | | | | | | |  | **40–60 years** | | | | | | | | |
| --- | --- | --- | --- | --- | --- | --- | --- | --- | --- | --- | --- | --- | --- | --- | --- | --- | --- | --- | --- |
|  | **ACL-injured** | | | | |  | **Healthy** | | |  | **ACL-injured** | | | | |  | **Healthy** | | |
|  | **female** | |  | **male** | |  | **female** |  | **male** |  | **female** | |  | **male** | |  | **Female** |  | **male** |
|  | **ACL_in** | **ACL_unin** |  | **ACL_in** | **ACL_unin** |  | **HEA_rand** |  | **HEA_rand** |  | **ACL_in** | **ACL_unin** |  | **ACL_in** | **ACL_unin** |  | **HEA_rand** |  | **HEA_rand** |
| **n** | 11 | 11 |  | 9 | 9 |  | 11 |  | 12 |  | 10 | 10 |  | 4 | 4 |  | 12 |  | 9 |
| **Compartments** | | | | | | | | | | | | | | | | | | | |
| Baseline FTJ.T | 35.2 ± 2.7 | 33.7 ± 2.3 |  | 32.1 ± 3.6 | 30.2 ± 2.2 |  | 33.1 ± 3.0 |  | 33.9 ± 2.4 |  | 35.9 ± 2.4 | 35.2 ± 3.5 |  | 34.2 ± 4.5 | 35.5 ± 7.4 |  | 34.7 ± 3.2 |  | 33.5 ± 4.1 |
| 2-year FTJ.T | 34.9 ± 4.3 | 34.7 ± 2.2 |  | 32.7 ± 4.0 | 31.1 ± 2.3 |  | 34.0 ± 2.5 |  | 34.1 ± 2.8 |  | 36.4 ± 2.7 | 36.0 ± 4.2 |  | 35.0 ± 2.5 | 34.6 ± 4.3 |  | 35.7 ± 2.7 |  | 33.9 ± 4.8 |
| Change FTJ.T | -0.3 ± 2.2 | 1.0 ± 1.5 |  | 0.7 ± 1.5 | 0.9 ± 1.2 |  | 0.9 ± 1.4 |  | 0.2 ± 1.5 |  | 0.5 ± 1.7 | 0.8 ± 1.8 |  | 0.8 ± 2.2 | -0.9 ± 3.6 |  | 1.0 ± 2.2 |  | 0.4 ± 1.2 |
| Baseline FTJ.D | 23.4 ± 1.9 | 21.3 ± 1.0 |  | 22.2 ± 1.9 | 19.8 ± 1.3 |  | 21.0 ± 1.5 |  | 21.2 ± 1.5 |  | 24.3 ± 1.9 | 22.1 ± 2.5 |  | 23.9 ± 2.6 | 23.4 ± 4.9 |  | 22.2 ± 2.8 |  | 21.2 ± 2.9 |
| 2-year FTJ.D | 23.2 ± 2.6 | 21.9 ± 1.1 |  | 23.1 ± 2.7 | 20.7 ± 1.5 |  | 21.4 ± 0.9 |  | 21.3 ± 1.8 |  | 24.6 ± 1.9 | 23.0 ± 2.2 |  | 24.6 ± 1.7 | 21.9 ± 2.2 |  | 22.7 ± 2.4 |  | 21.3 ± 2.7 |
| Change FTJ.D | -0.2 ± 1.2 | 0.6 ± 1.1 |  | 0.9 ± 1.2 | 1.0 ± 0.9 |  | 0.4 ± 1.2 |  | 0.0 ± 0.5 |  | 0.3 ± 1.3 | 0.9 ± 1.7 |  | 0.8 ± 1.2 | -1.5 ± 3.3 |  | 0.5 ± 1.1 |  | 0.1 ± 0.6 |
| Baseline FTJ.S | 45.5 ± 3.9 | 44.4 ± 3.9 |  | 40.9 ± 5.2 | 39.5 ± 3.2 |  | 43.6 ± 4.8 |  | 45.1 ± 3.7 |  | 46.0 ± 3.6 | 46.5 ± 5.0 |  | 43.4 ± 7.1 | 46.2 ± 9.7 |  | 45.5 ± 3.9 |  | 44.4 ± 6.1 |
| 2-year FTJ.S | 45.0 ± 5.9 | 45.7 ± 3.5 |  | 41.4 ± 5.4 | 40.3 ± 3.3 |  | 44.8 ± 4.2 |  | 45.5 ± 4.2 |  | 46.6 ± 4.6 | 47.3 ± 6.2 |  | 44.3 ± 4.1 | 45.8 ± 6.6 |  | 46.9 ± 3.5 |  | 45.1 ± 7.3 |
| Change FTJ.S | -0.5 ± 3.1 | 1.4 ± 2.4 |  | 0.4 ± 1.9 | 0.8 ± 1.6 |  | 1.3 ± 1.6 |  | 0.3 ± 2.6 |  | 0.6 ± 2.7 | 0.7 ± 2.5 |  | 0.9 ± 3.2 | -0.4 ± 4.1 |  | 1.4 ± 3.3 |  | 0.7 ± 1.8 |
| Baseline MFTC.T | 34.2 ± 4.0 | 33.3 ± 2.7 |  | 32.0 ± 3.7 | 29.3 ± 3.1 |  | 33.5 ± 3.1 |  | 33.9 ± 3.8 |  | 35.2 ± 4.7 | 34.7 ± 4.2 |  | 33.6 ± 4.4 | 37.9 ± 9.5 |  | 34.3 ± 3.2 |  | 34.4 ± 5.2 |
| 2-year MFTC.T | 33.7 ± 5.1 | 33.9 ± 2.4 |  | 32.1 ± 3.7 | 30.1 ± 3.2 |  | 34.1 ± 2.6 |  | 33.8 ± 3.9 |  | 36.5 ± 5.5 | 35.3 ± 5.0 |  | 34.1 ± 3.3 | 36.6 ± 5.7 |  | 35.5 ± 3.0 |  | 34.8 ± 6.2 |
| Change MFTC.T | -0.5 ± 2.3 | 0.6 ± 1.6 |  | 0.2 ± 1.5 | 0.7 ± 1.0 |  | 0.6 ± 1.4 |  | 0.0 ± 1.5 |  | 1.4 ± 2.9 | 0.6 ± 1.9 |  | 0.5 ± 2.2 | -1.3 ± 4.4 |  | 1.2 ± 2.5 |  | 0.4 ± 1.5 |
| Baseline MFTC.D | 22.7 ± 2.4 | 20.9 ± 0.8 |  | 22.3 ± 1.5 | 19.7 ± 1.6 |  | 21.0 ± 1.9 |  | 21.2 ± 2.3 |  | 23.4 ± 2.5 | 21.5 ± 2.5 |  | 23.2 ± 2.2 | 24.5 ± 5.7 |  | 22.1 ± 2.6 |  | 21.6 ± 3.3 |
| 2-year MFTC.D | 22.6 ± 2.7 | 21.3 ± 1.4 |  | 22.8 ± 2.1 | 20.7 ± 2.0 |  | 21.1 ± 1.0 |  | 21.3 ± 2.5 |  | 24.2 ± 2.7 | 22.7 ± 2.6 |  | 23.8 ± 1.8 | 22.8 ± 2.0 |  | 22.7 ± 2.4 |  | 21.8 ± 3.2 |
| Change MFTC.D | -0.1 ± 0.7 | 0.4 ± 1.0 |  | 0.6 ± 1.0 | 1.0 ± 0.9 |  | 0.1 ± 1.6 |  | 0.1 ± 0.6 |  | 0.8 ± 1.5 | 1.1 ± 1.6 |  | 0.7 ± 1.1 | -1.6 ± 3.9 |  | 0.6 ± 1.2 |  | 0.2 ± 0.7 |
| Baseline MFTC.S | 44.3 ± 5.9 | 44.0 ± 4.8 |  | 40.6 ± 5.9 | 37.8 ± 4.8 |  | 44.3 ± 5.0 |  | 44.9 ± 5.5 |  | 45.4 ± 7.1 | 45.9 ± 6.2 |  | 42.9 ± 7.5 | 49.7 ± 13.0 |  | 44.7 ± 4.3 |  | 45.7 ± 7.8 |
| 2-year MFTC.S | 43.3 ± 7.5 | 44.7 ± 4.1 |  | 40.4 ± 5.5 | 38.3 ± 4.4 |  | 45.2 ± 4.5 |  | 44.8 ± 5.5 |  | 47.3 ± 8.9 | 46.1 ± 7.7 |  | 43.3 ± 5.8 | 48.8 ± 9.1 |  | 46.4 ± 4.5 |  | 46.2 ± 9.6 |
| Change MFTC.S | -0.9 ± 3.9 | 0.7 ± 2.7 |  | -0.2 ± 2.2 | 0.5 ± 1.6 |  | 0.9 ± 1.7 |  | -0.2 ± 2.4 |  | 1.9 ± 4.7 | 0.2 ± 3.0 |  | 0.4 ± 3.1 | -1.0 ± 5.0 |  | 1.7 ± 3.7 |  | 0.5 ± 2.3 |
| Baseline LFTC.T | 36.1 ± 2.4 | 34.0 ± 2.5 |  | 32.2 ± 3.9 | 31.1 ± 1.7 |  | 32.7 ± 3.2 |  | 34.0 ± 1.8 |  | 36.7 ± 1.5 | 35.8 ± 3.6 |  | 34.7 ± 4.7 | 33.1 ± 5.6 |  | 35.0 ± 3.6 |  | 32.7 ± 3.2 |
| 2-year LFTC.T | 36.0 ± 4.4 | 35.4 ± 2.5 |  | 33.3 ± 4.6 | 32.2 ± 2.1 |  | 33.9 ± 2.4 |  | 34.5 ± 2.2 |  | 36.2 ± 1.0 | 36.8 ± 4.2 |  | 35.9 ± 2.4 | 32.6 ± 3.4 |  | 35.9 ± 2.9 |  | 33.1 ± 3.5 |
| Change LFTC.T | -0.1 ± 2.8 | 1.5 ± 1.7 |  | 1.2 ± 1.6 | 1.0 ± 1.8 |  | 1.2 ± 1.6 |  | 0.4 ± 1.9 |  | -0.5 ± 1.0 | 1.0 ± 2.3 |  | 1.1 ± 2.5 | -0.5 ± 3.0 |  | 0.9 ± 2.1 |  | 0.4 ± 1.3 |
| Baseline LFTC.D | 24.1 ± 1.8 | 21.6 ± 1.6 |  | 22.1 ± 2.4 | 19.9 ± 1.3 |  | 21.0 ± 1.6 |  | 21.2 ± 1.0 |  | 25.3 ± 2.0 | 22.7 ± 2.7 |  | 24.6 ± 3.0 | 22.4 ± 4.3 |  | 22.2 ± 3.0 |  | 20.9 ± 2.6 |
| 2-year LFTC.D | 23.8 ± 3.1 | 22.4 ± 1.1 |  | 23.3 ± 3.3 | 20.8 ± 1.5 |  | 21.8 ± 1.5 |  | 21.2 ± 1.2 |  | 25.1 ± 1.9 | 23.3 ± 2.2 |  | 25.4 ± 1.9 | 21.1 ± 2.6 |  | 22.6 ± 2.4 |  | 20.8 ± 2.3 |
| Change LFTC.D | -0.3 ± 2.0 | 0.8 ± 1.5 |  | 1.2 ± 1.5 | 0.9 ± 1.3 |  | 0.7 ± 1.0 |  | 0.0 ± 0.6 |  | -0.2 ± 1.5 | 0.6 ± 2.0 |  | 0.9 ± 1.4 | -1.4 ± 2.7 |  | 0.5 ± 1.0 |  | -0.1 ± 0.8 |
| Baseline LFTC.S | 46.7 ± 3.8 | 44.8 ± 4.1 |  | 41.2 ± 5.3 | 41.2 ± 2.4 |  | 42.8 ± 5.2 |  | 45.4 ± 3.0 |  | 46.7 ± 2.9 | 47.1 ± 5.2 |  | 43.9 ± 6.8 | 42.6 ± 6.7 |  | 46.3 ± 4.2 |  | 43.2 ± 4.7 |
| 2-year LFTC.S | 46.7 ± 5.9 | 46.8 ± 4.1 |  | 42.3 ± 6.0 | 42.3 ± 3.0 |  | 44.5 ± 4.3 |  | 46.2 ± 4.2 |  | 45.9 ± 2.3 | 48.5 ± 6.4 |  | 45.3 ± 3.2 | 42.9 ± 4.5 |  | 47.4 ± 3.7 |  | 44.1 ± 5.4 |
| Change LFTC.S | 0.0 ± 3.6 | 2.0 ± 2.7 |  | 1.1 ± 1.9 | 1.1 ± 2.5 |  | 1.6 ± 2.4 |  | 0.8 ± 3.5 |  | -0.8 ± 2.3 | 1.3 ± 3.2 |  | 1.4 ± 3.8 | 0.3 ± 3.2 |  | 1.2 ± 3.3 |  | 0.9 ± 2.1 |
| **Regions** | | | | | | | | | | | | | | | | | | | |
| Baseline MT.T | 32.3 ± 3.5 | 30.2 ± 2.7 |  | 29.2 ± 3.5 | 26.6 ± 3.4 |  | 29.4 ± 3.1 |  | 31.3 ± 3.9 |  | 31.3 ± 4.4 | 30.6 ± 3.2 |  | 29.9 ± 4.1 | 31.3 ± 5.4 |  | 30.3 ± 2.9 |  | 29.9 ± 3.7 |
| 2-year MT.T | 32.2 ± 4.9 | 31.0 ± 2.9 |  | 29.7 ± 3.9 | 27.4 ± 2.9 |  | 29.8 ± 2.6 |  | 31.1 ± 3.9 |  | 32.4 ± 5.1 | 30.8 ± 3.7 |  | 30.2 ± 2.7 | 31.2 ± 4.4 |  | 30.7 ± 3.1 |  | 29.8 ± 4.7 |
| Change MT.T | -0.1 ± 3.0 | 0.8 ± 1.2 |  | 0.5 ± 2.2 | 0.8 ± 1.3 |  | 0.5 ± 2.0 |  | -0.2 ± 1.7 |  | 1.1 ± 2.9 | 0.2 ± 1.8 |  | 0.2 ± 1.9 | -0.1 ± 2.5 |  | 0.4 ± 2.3 |  | -0.1 ± 1.7 |
| Baseline MT.D | 20.6 ± 2.1 | 18.9 ± 1.1 |  | 20.1 ± 2.4 | 17.7 ± 2.1 |  | 18.1 ± 1.3 |  | 19.5 ± 2.8 |  | 19.9 ± 2.5 | 18.7 ± 1.9 |  | 20.0 ± 2.0 | 19.8 ± 2.7 |  | 19.6 ± 2.5 |  | 18.6 ± 3.2 |
| 2-year MT.D | 21.1 ± 2.5 | 19.6 ± 1.4 |  | 20.7 ± 3.3 | 18.6 ± 2.2 |  | 18.6 ± 1.7 |  | 19.6 ± 3.0 |  | 20.7 ± 2.1 | 19.3 ± 2.0 |  | 20.6 ± 1.7 | 19.5 ± 1.4 |  | 20.0 ± 2.2 |  | 18.8 ± 3.2 |
| Change MT.D | 0.4 ± 1.2 | 0.6 ± 1.3 |  | 0.6 ± 1.7 | 0.9 ± 1.0 |  | 0.4 ± 1.2 |  | 0.1 ± 0.8 |  | 0.8 ± 1.3 | 0.7 ± 0.9 |  | 0.6 ± 0.5 | -0.3 ± 1.4 |  | 0.3 ± 1.1 |  | 0.2 ± 0.8 |
| Baseline MT.S | 42.7 ± 5.3 | 40.2 ± 4.3 |  | 37.5 ± 5.0 | 34.7 ± 4.9 |  | 39.2 ± 5.6 |  | 41.9 ± 5.4 |  | 41.5 ± 7.0 | 41.2 ± 4.9 |  | 39.0 ± 7.5 | 41.9 ± 8.2 |  | 39.7 ± 3.8 |  | 40.4 ± 5.3 |
| 2-year MT.S | 42.1 ± 7.5 | 41.1 ± 4.8 |  | 37.9 ± 5.1 | 35.4 ± 4.0 |  | 39.7 ± 4.5 |  | 41.4 ± 5.7 |  | 43.0 ± 8.8 | 40.9 ± 6.0 |  | 39.0 ± 5.3 | 41.9 ± 7.3 |  | 40.1 ± 4.7 |  | 40.0 ± 7.1 |
| Change MT.S | -0.6 ± 5.1 | 0.9 ± 2.1 |  | 0.4 ± 3.0 | 0.7 ± 1.8 |  | 0.4 ± 3.0 |  | -0.5 ± 2.7 |  | 1.5 ± 4.4 | -0.3 ± 3.0 |  | 0.0 ± 3.1 | 0.0 ± 3.5 |  | 0.3 ± 3.5 |  | -0.4 ± 2.8 |
| Baseline cMF.T | 36.2 ± 5.7 | 36.4 ± 4.4 |  | 34.8 ± 4.7 | 32.0 ± 3.4 |  | 37.7 ± 4.0 |  | 36.4 ± 4.1 |  | 39.0 ± 5.5 | 38.8 ± 6.1 |  | 37.3 ± 4.7 | 44.5 ± 13.7 |  | 38.3 ± 5.3 |  | 38.9 ± 7.4 |
| 2-year cMF.T | 35.2 ± 6.4 | 36.9 ± 3.9 |  | 34.6 ± 5.1 | 32.7 ± 4.1 |  | 38.4 ± 4.1 |  | 36.6 ± 4.7 |  | 40.6 ± 6.3 | 39.8 ± 6.8 |  | 38.1 ± 3.9 | 42.1 ± 7.7 |  | 40.4 ± 5.6 |  | 39.7 ± 8.5 |
| Change cMF.T | -1.0 ± 2.3 | 0.4 ± 2.4 |  | -0.2 ± 1.2 | 0.7 ± 1.5 |  | 0.6 ± 1.7 |  | 0.2 ± 2.4 |  | 1.6 ± 3.2 | 1.1 ± 2.3 |  | 0.8 ± 2.6 | -2.4 ± 6.7 |  | 2.0 ± 3.8 |  | 0.8 ± 1.8 |
| Baseline cMF.D | 24.8 ± 3.2 | 23.0 ± 1.3 |  | 24.4 ± 1.3 | 21.7 ± 1.6 |  | 23.9 ± 3.4 |  | 22.8 ± 2.3 |  | 26.9 ± 3.2 | 24.4 ± 3.8 |  | 26.3 ± 2.5 | 29.1 ± 8.9 |  | 24.6 ± 3.6 |  | 24.6 ± 3.9 |
| 2-year cMF.D | 24.1 ± 3.4 | 23.1 ± 1.8 |  | 24.9 ± 2.4 | 22.8 ± 2.7 |  | 23.7 ± 2.2 |  | 23.0 ± 2.7 |  | 27.6 ± 3.6 | 26.0 ± 4.3 |  | 27.0 ± 2.1 | 26.2 ± 2.7 |  | 25.4 ± 3.3 |  | 24.8 ± 3.8 |
| Change cMF.D | -0.7 ± 1.3 | 0.1 ± 1.3 |  | 0.5 ± 1.4 | 1.1 ± 1.8 |  | -0.2 ± 2.3 |  | 0.2 ± 0.9 |  | 0.8 ± 2.4 | 1.6 ± 3.3 |  | 0.8 ± 1.9 | -3.0 ± 6.9 |  | 0.8 ± 1.8 |  | 0.2 ± 0.9 |
| Baseline cMF.S | 45.8 ± 8.3 | 47.8 ± 7.6 |  | 43.7 ± 7.6 | 40.8 ± 5.6 |  | 49.3 ± 5.7 |  | 48.0 ± 6.2 |  | 49.3 ± 7.9 | 50.7 ± 8.9 |  | 46.7 ± 7.7 | 57.6 ± 18.3 |  | 49.6 ± 7.4 |  | 51.0 ± 11.2 |
| 2-year cMF.S | 44.6 ± 9.1 | 48.3 ± 6.9 |  | 43.0 ± 7.5 | 41.2 ± 5.4 |  | 50.6 ± 6.4 |  | 48.1 ± 6.7 |  | 51.6 ± 9.8 | 51.3 ± 10.1 |  | 47.6 ± 6.3 | 55.6 ± 12.3 |  | 52.7 ± 8.3 |  | 52.4 ± 13.1 |
| Change cMF.S | -1.3 ± 3.6 | 0.5 ± 3.8 |  | -0.8 ± 1.8 | 0.4 ± 2.3 |  | 1.3 ± 1.9 |  | 0.1 ± 4.0 |  | 2.3 ± 5.4 | 0.7 ± 3.6 |  | 0.8 ± 3.4 | -2.0 ± 6.9 |  | 3.1 ± 5.6 |  | 1.4 ± 2.9 |
| Baseline LT.T | 31.0 ± 1.9 | 30.1 ± 2.1 |  | 28.3 ± 3.1 | 27.7 ± 2.1 |  | 28.4 ± 2.4 |  | 29.5 ± 1.3 |  | 31.8 ± 3.0 | 31.6 ± 2.9 |  | 31.5 ± 5.6 | 29.4 ± 6.4 |  | 32.4 ± 4.0 |  | 29.8 ± 3.6 |
| 2-year LT.T | 30.6 ± 3.1 | 31.3 ± 2.3 |  | 29.5 ± 4.4 | 29.5 ± 2.4 |  | 29.6 ± 1.9 |  | 30.0 ± 1.8 |  | 32.1 ± 2.6 | 32.4 ± 2.9 |  | 32.6 ± 2.9 | 29.5 ± 5.4 |  | 32.7 ± 3.2 |  | 30.0 ± 4.2 |
| Change LT.T | -0.4 ± 2.7 | 1.2 ± 1.8 |  | 1.3 ± 2.1 | 1.8 ± 1.8 |  | 1.2 ± 1.4 |  | 0.5 ± 1.4 |  | 0.3 ± 2.1 | 0.8 ± 2.5 |  | 1.1 ± 2.7 | 0.1 ± 1.8 |  | 0.3 ± 2.5 |  | 0.2 ± 1.4 |
| Baseline LT.D | 22.0 ± 2.1 | 20.7 ± 2.8 |  | 19.5 ± 2.2 | 18.2 ± 1.2 |  | 19.5 ± 1.3 |  | 19.5 ± 1.3 |  | 23.0 ± 2.6 | 21.4 ± 2.1 |  | 23.6 ± 4.1 | 20.5 ± 4.1 |  | 21.3 ± 3.3 |  | 19.7 ± 3.2 |
| 2-year LT.D | 21.8 ± 2.6 | 21.3 ± 1.4 |  | 21.3 ± 3.9 | 19.6 ± 1.4 |  | 20.4 ± 1.8 |  | 19.5 ± 1.4 |  | 23.1 ± 2.2 | 22.0 ± 2.1 |  | 24.2 ± 3.0 | 20.1 ± 3.4 |  | 22.0 ± 3.1 |  | 19.7 ± 3.4 |
| Change LT.D | -0.2 ± 2.4 | 0.5 ± 2.4 |  | 1.8 ± 1.9 | 1.4 ± 1.4 |  | 0.9 ± 1.2 |  | 0.0 ± 0.8 |  | 0.1 ± 2.2 | 0.6 ± 1.6 |  | 0.6 ± 1.1 | -0.4 ± 1.5 |  | 0.6 ± 1.1 |  | 0.0 ± 0.8 |
| Baseline LT.S | 39.4 ± 3.2 | 38.6 ± 2.7 |  | 36.4 ± 4.2 | 36.5 ± 3.0 |  | 36.5 ± 4.5 |  | 38.8 ± 2.6 |  | 40.0 ± 5.0 | 40.9 ± 4.7 |  | 38.9 ± 7.9 | 37.7 ± 8.7 |  | 42.4 ± 5.0 |  | 39.2 ± 4.9 |
| 2-year LT.S | 38.7 ± 4.3 | 40.5 ± 3.5 |  | 37.2 ± 5.2 | 38.6 ± 3.7 |  | 37.9 ± 3.7 |  | 39.7 ± 3.3 |  | 40.4 ± 4.3 | 41.9 ± 4.9 |  | 40.4 ± 3.6 | 38.2 ± 7.5 |  | 42.5 ± 3.7 |  | 39.6 ± 5.6 |
| Change LT.S | -0.6 ± 3.3 | 1.9 ± 2.1 |  | 0.8 ± 2.7 | 2.2 ± 2.5 |  | 1.5 ± 1.9 |  | 0.9 ± 2.4 |  | 0.4 ± 3.0 | 0.9 ± 3.9 |  | 1.5 ± 4.3 | 0.5 ± 2.1 |  | 0.0 ± 3.9 |  | 0.4 ± 2.2 |
| Baseline cLF.T | 41.2 ± 4.6 | 37.9 ± 4.1 |  | 36.0 ± 5.1 | 34.6 ± 2.6 |  | 37.0 ± 4.4 |  | 38.5 ± 3.5 |  | 41.5 ± 3.1 | 40.0 ± 5.5 |  | 38.0 ± 3.8 | 36.7 ± 5.0 |  | 37.7 ± 4.0 |  | 35.6 ± 3.6 |
| 2-year cLF.T | 41.4 ± 6.6 | 39.5 ± 3.8 |  | 37.1 ± 5.7 | 34.8 ± 2.1 |  | 38.3 ± 3.6 |  | 38.9 ± 3.7 |  | 40.3 ± 1.6 | 41.2 ± 6.5 |  | 39.2 ± 2.1 | 35.7 ± 2.8 |  | 39.2 ± 3.9 |  | 36.2 ± 4.6 |
| Change cLF.T | 0.2 ± 3.4 | 1.7 ± 3.2 |  | 1.1 ± 1.4 | 0.2 ± 2.3 |  | 1.3 ± 2.6 |  | 0.4 ± 2.9 |  | -1.3 ± 2.2 | 1.2 ± 2.9 |  | 1.2 ± 2.4 | -1.1 ± 4.3 |  | 1.5 ± 2.5 |  | 0.7 ± 2.3 |
| Baseline cLF.D | 26.2 ± 3.2 | 22.5 ± 1.8 |  | 24.7 ± 3.2 | 21.5 ± 1.8 |  | 22.5 ± 2.4 |  | 23.0 ± 1.6 |  | 27.6 ± 2.8 | 24.1 ± 4.0 |  | 25.5 ± 1.9 | 24.4 ± 4.6 |  | 23.0 ± 3.5 |  | 22.1 ± 2.5 |
| 2-year cLF.D | 25.9 ± 4.5 | 23.6 ± 1.8 |  | 25.4 ± 3.4 | 22.0 ± 1.9 |  | 23.1 ± 2.4 |  | 22.9 ± 1.6 |  | 27.1 ± 2.9 | 24.7 ± 2.9 |  | 26.6 ± 1.1 | 22.1 ± 2.4 |  | 23.3 ± 3.0 |  | 22.0 ± 2.1 |
| Change cLF.D | -0.3 ± 2.1 | 1.0 ± 1.4 |  | 0.7 ± 1.2 | 0.5 ± 1.9 |  | 0.5 ± 1.4 |  | -0.1 ± 0.8 |  | -0.5 ± 2.5 | 0.6 ± 3.2 |  | 1.1 ± 1.6 | -2.3 ± 4.1 |  | 0.3 ± 1.9 |  | -0.1 ± 1.1 |
| Baseline cLF.S | 54.0 ± 6.6 | 50.9 ± 6.3 |  | 46.0 ± 6.9 | 46.0 ± 3.6 |  | 49.2 ± 6.6 |  | 51.9 ± 5.6 |  | 53.4 ± 5.1 | 53.3 ± 7.6 |  | 49.0 ± 5.8 | 47.5 ± 5.5 |  | 50.1 ± 4.7 |  | 47.2 ± 5.5 |
| 2-year cLF.S | 54.7 ± 8.8 | 53.1 ± 6.4 |  | 47.5 ± 7.9 | 45.9 ± 3.1 |  | 51.0 ± 5.7 |  | 52.7 ± 6.6 |  | 51.4 ± 3.2 | 55.0 ± 10.0 |  | 50.3 ± 3.6 | 47.5 ± 3.5 |  | 52.4 ± 5.2 |  | 48.5 ± 7.8 |
| Change cLF.S | 0.6 ± 4.7 | 2.1 ± 5.5 |  | 1.4 ± 2.3 | -0.1 ± 3.0 |  | 1.8 ± 4.2 |  | 0.8 ± 5.4 |  | -2.0 ± 3.9 | 1.7 ± 4.1 |  | 1.3 ± 3.3 | 0.0 ± 4.4 |  | 2.3 ± 4.1 |  | 1.4 ± 3.7 |

ACL—anterior cruciate ligament; ACL_in—ACL-injured; ACL_unin—ACL-uninjured; HEA—healthy; FTJ—femorotibial joint; T—total T2; D—deep zone T2; S—superficial zone T2; MFTC—medial femorotibial compartment; LFTC—lateral femorotibial compartment; MT—medial tibia; cMF—central medial femur; LT—lateral tibia; cLF—central lateral femur
